# Prognostic Modelling Studies of Coronary Heart Disease—A Systematic Review of Conventional and Genetic Risk Factor Studies

**DOI:** 10.3390/jcdd9090295

**Published:** 2022-09-05

**Authors:** Nayla Nasr, Beáta Soltész, János Sándor, Róza Adány, Szilvia Fiatal

**Affiliations:** 1 Doctoral School of Health Sciences, University of Debrecen, 4032 Debrecen, Hungary; 2 Department of Public Health and Epidemiology, Faculty of Medicine, University of Debrecen, 4032 Debrecen, Hungary; 3 Faculty of Public Health, University of Debrecen, 4032 Debrecen, Hungary

**Keywords:** systematic review, coronary heart disease, prognostic models, genetic risk factors, conventional risk factors

## Abstract

This study aims to provide an overview of multivariable prognostic modelling studies developed for coronary heart disease (CHD) in the general population and to explore the optimal prognostic model by comparing the models’ performance. A systematic review was performed using Embase, PubMed, Cochrane, Web of Science, and Scopus databases until 30 November 2019. In this work, only prognostic studies describing conventional risk factors alone or a combination of conventional and genomic risk factors, being developmental and/or validation prognostic studies of a multivariable model, were included. A total of 4021 records were screened by titles and abstracts, and 72 articles were eligible. All the relevant studies were checked by comparing the discrimination, reclassification, and calibration measures. Most of the models were developed in the United States and Canada and targeted the general population. The models included a set of similar predictors, such as age, sex, smoking, cholesterol level, blood pressure, BMI, and diabetes mellitus. In this study, many articles were identified and screened for consistency and reliability using CHARM and GRIPS statements. However, the usefulness of most prognostic models was not demonstrated; only a limited number of these models supported clinical evidence. Unfortunately, substantial heterogeneity was recognized in the definition and outcome of CHD events. The inclusion of genetic risk scores in addition to conventional risk factors might help in predicting the incidence of CHDs; however, the generalizability of the existing prognostic models remains open. Validation studies for the existing developmental models are needed to ensure generalizability, improve the research quality, and increase the transparency of the study.

## 1. Introduction

Coronary heart disease (CHD) is a leading cause of morbidity, mortality, and disability in developed and developing countries [1,2]. The WHO estimates that by 2030, the number of annual deaths caused by cardiovascular diseases (mainly from CHD and stroke) will reach almost 23.6 million [3,4]. CHD is the greatest cause of mortality and loss of disability-adjusted life years (DALYs) worldwide, accounting for 7 million deaths and 129 million DALYs annually [5]. A wide range of preventive interventions for CHD are available for high-risk individuals through effective medication and comprehensive modification of risk factors such as elevated LDL cholesterol levels, heavy smoking, unhealthy diet, and physical inactivity [6,7,8,9,10]. Both types of interventions can be significantly improved by accurate risk assessment [7]. Accurate risk identification allows medical professionals to intervene in managing risk factors prior to the onset of more critical conditions, thereby improving the quality of life of the patient [11]. Accurate risk assessments can improve their effectiveness. Accurate risk identification allows medical professionals to intervene early before the developments of the diseases, thus improving the quality of life of the patient [12,13]. Risk assessment of CHD requires thoroughness, completeness, and accuracy in obtaining information and measurements for identifying subgroups with elevated risk and predicting the timing of disease onset [13,14].

Prognostic models are used to estimate the probability of developing a particular outcome in the future with the aim of assisting clinicians in disease prediction and enhancing informed decision-making with the patient [11,12,13,14,15]. These models, in general, use two types of performance measures: discrimination (the ability of the model to distinguish individuals who develop events from those who do not) and calibration (how accurately the model prediction matches the overall observed event rates) [16]. Prognostic models are more likely to be reliable and useful in practice when they are developed using a large, high-quality data set, based on a study protocol with a sound statistical analysis plan, and externally validated by using independent data sets [17]. Despite the importance of predicting future CHD among initially healthy adults, the predictive accuracy of the models has often seemed disappointing because most individuals who eventually suffer a cardiovascular disease event were previously at average risk rather than high risk [18]. Data from the cohort (retrospective and prospective), nested case-control, or case-cohort studies are recommended for prognostic modelling studies (developmental and/or validation) [15].

Over the past year, abundant prognostic models have been developed to estimate the risk of developing cardiovascular diseases, such as the Framingham, SCORE, QRISK, QRISK2, and ASSIGN models [10,19,20,21]. Most of the existing models are based on the Framingham model [20]. The original Framingham model included age, sex, LDL cholesterol, HDL cholesterol levels, blood pressure level, hypertension medication, smoking, and diabetes mellitus [2,3,4,5,6,7,8,9,10,20,21]. Different markers were then added to this model as a response to deficiencies in improving performance, such as coronary artery calcification score, C-reactive protein, fibrinogen, homocysteine, and apolipoprotein [22,23,24,25,26,27,28]. Previous studies found that all three models based on the Framingham score—the Framingham Adult Treatment Panel (ATP) III model, the Framingham Wilson model, and pooled cohort equations (PCE)—provide an incomplete prognosis of CHD events [29]. Framingham functions have overestimated the CHD risk in some populations (British and European natives), leading to a concern that it may not be appropriate for other populations [30,31,32]. However, two problems remain: first, there is no consensus about the most suitable and optimal model for predicting CHD in the general population, and second, it is not clear which biomarkers or event genetic markers should be incorporated in the risk model in addition to conventional factors.

For any novel CHD risk factor to be useful in a clinical setting, it must significantly enhance event prognosis based on easily measurable conventional risk factors (CRFs) such as age, cholesterol level, blood pressure, or body mass index; thus, any such factor(s) must have a major impact on risk [13,14].

It is now universally accepted that CHD risk is known to be modified by interaction of both multiple genetics and environmental components [33]. Genome-wide association studies (GWAS) have so far identified a hundred loci associated with many cardiovascular diseases and traits [34]. Out of these, more than 97 single-nucleotide polymorphisms (SNPs) have been associated with CHD risk and myocardial infarction [34,35,36,37,38,39].

CHD risk stratification for primary prevention based only on conventional risk factors seems to be less than efficient. The genetic risk score computed from the recently discovered genetic variants might offer a potential solution in cardiovascular primary prevention. It was found that GRS-based risk stratification performed on large populations, followed by lifestyle changes (e.g., physical activity, diet) or statin therapy, are associated with a significant 40% to 50% reduction in cardiac events in the high-genetic-risk group [40,41,42]. Formerly, the association between some SNPs (e.g., gene encoding for ion-channel subunits, and in coronary blood flow regulation), and coronary microvascular function independently from coronary artery disease was defined; specifically, the role of adenosine triphosphate-sensitive potassium channels (ATP), which are the end effectors of several regulatory mechanisms for coronary flow reserves [43].

Recognizing the need for larger studies, we performed a systematic review to provide an overview of multivariable prognostic models developed to predict the risk of CHD in the general population, to find the optimal models by assessing their performance in estimating CHD risk and to provide researchers with prognostic models by describing the optimal combination of predictors, including conventional risk factors, genetic risk scores, and biomarkers. We hypothesized that adding the genetic risk score to conventional risk-factor-based models would improve the ability of these models to predict CHD events in the general population.

## 2. Materials and Methods

Our present study was registered in PROSPERO (ID: CRD42021234224). We conducted our systematic review based on the PRISMA guidelines by following the recently published Cochrane Prognosis Methods Group guidelines [44] by using the Checklist for critical Appraisal and data extraction for the systematic review of prediction Modelling Studies (CHARMS) statement for assessing the quality of the prognostic modelling studies [15]. The Genetic Risk Prediction Studies (GRIPS) Statement was used to assess genetic prognostic modelling studies [45]. We used five databases—Embase, PubMed, Cochrane, Web of Science, and Scopus—and we applied a human filter on 30 November 2019 to identify original articles of the developmental and/or validation of prognostic models describing the combination of conventional and genomic risk factors for incident CHD. We searched the databases using the following key search terms: (“validation” OR “prediction” OR “predict” OR “risk” OR “prognosis”) AND (“ROC” OR “area under the curve” OR “c-statistic” OR “c statistic” OR “discrimination” OR “discriminate”) AND (“coronary heart disease” OR “CHD” OR “coronary disease”). The detailed protocol can be found in the Supplementary Material (see pages 1–2).

## 3. Eligibility Criteria

We included all original articles describing the estimation of risk associated with CHD morbidity or mortality in individuals, developmental and/or validation modelling studies (internal/external), and the models’ performance for predicting CHD in the general population (performance measures regarding calibration, discrimination, and reclassification are available). Two study designs were included: (nested) case-control and cohort. Articles describing clinical models with intervention (treatment) and studies describing the prediction models of CHD in individuals with certain health conditions, such as HIV, HBV, diabetes, and kidney failure, were excluded.

## 4. Selection Process

**Figure 1:**
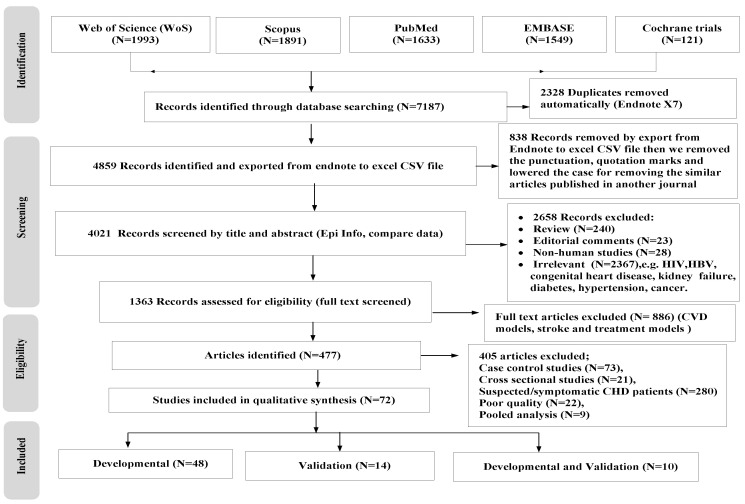
Flow chart of the selection process of coronary heart disease risk prognostic models.

Initially, two reviewers (N.M.G and O.A.) independently screened the titles and abstracts of all studies identified according to the keywords and inclusion criteria, and then duplicates were removed. After consensus, full-text articles were then obtained and examined for quality. If there was any disagreement regarding the article’s inclusion, a third-party evaluation was performed to reach a consensus. We compared the work of the reviewers using the Epi Info7 program developed by the Centre’s for Disease Control and Prevention (CDC) to minimize biases. The Preferred Reporting Items for Systematic Review and Meta-Analysis (PRISMA) flow chart summarizes the selection process (Figure 1).

We categorized the eligible full-text articles into three groups: (1) Developmental studies—such models commonly aim to identify important predictors by selecting predictors, combining them into a multivariable model, and then developing a final model and quantifying the predictive performance and validating this model internally using forms such as bootstrapping or cross-validation; (2) validation studies with or without updating such a model, aimed at assessing and comparing the predictive performance of an existing prognostic model using new participant data that were not used to develop the prognostic model and possibly adjust or update the model in case of poor performance based on the validation data; and (3) developmental studies with external validation in independent data [15].

## 5. Data Extraction and Critical Appraisal

The list of extracted items was based on the CHARMS and GRIPS Statement for reviewing the prognostic modelling of conventional and genetic studies [44,45]. The full list of extracted articles is available in the Supplementary Material (see references list of the included studies in the Supplementary Material from page 23).

## 6. Results

The search strategy identified 7187 potential articles; 2328 duplicates were removed automatically by Endnote X7 software, and 838 articles were removed after exporting the Endnote file to the CSV file (to create a new output style, removing the punctuation, lowering the case, and sorting the file). A total of 2658 articles were excluded based on title and abstract not being related to conventional or/and genetic risk modelling of CHD (reviews, editorial comments, and nonhuman and irrelevant studies) or prognostic modelling studies with subjects having comorbidities (HIV, HBV, diabetes mellitus, congenital heart disease, Chagas heart disease, and kidney failure). In total, 477 full texts were included after the exclusion of 405 other studies, such as case-control studies, cross-sectional studies, poor-quality studies, pooled analyses, and diagnostic/prognostic modelling studies on symptomatic or suspected patients (see Supplementary Table S1 for details). Finally, 72 eligible articles were included in this review (Figure 1, Tables S8 and S9 include the full list of the studies) [6,23,24,25,26,27,32,46,47,48,49,50,51,52,53,54,55,56,57,58,59,60,61,62,63,64,65,66,67,68,69,70,71,72,73,74,75,76,77,78,79,80,81,82,83,84,85,86,87,88,89,90,91,92,93,94,95,96,97,98,99,100,101,102,103,104,105,106,107,108,109]. We identified (*n* = 48) articles concerning the developmental CHD risk prognostic models; 14 articles described the external validation of the models, and 10 articles described the combinations of developmental and external validation (Figure 1, Supplementary Figure S3) The description of study populations, settings, periods of recruitment, length of follow-up, and methods of data collection of the reviewed model can be found in Supplementary Table S2.

**Figure 2:**
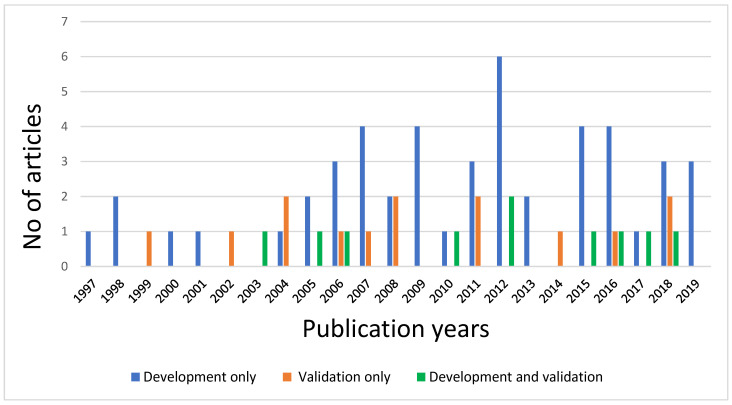
Numbers of publications on prognostic models included per year.

The number of developmental modelling studies (including genetic risk models) increased over the period 1997–2020, while the number of external validation and developmental validation modelling studies declined (Figure 2).

### 6.1. Studies Describing the Developmental Type of CHD Prognostic Models

#### 6.1.1. Frequency of Models, Study Designs, and Study Populations

In general, 58 articles (developmental plus developmental validation) described more than 157 different models (based on Framingham models plus the novel models created by the researchers). Most of the prognostic models (*n* = 68, 5%) were developed using data from cohort studies. Most of the models originated in the United States and Canada (*n* = 32, 45%) or Europe (*n* = 26, 36%); few studies originated from Asia (*n* = 14, 19%), and no developmental modelling studies originated from African countries. Framingham risk models developed for the US population were used multiple times to derive a novel model for different populations and countries. The model developed by Framingham, Wilson, and D’Agostino was used in 30 articles, and Framingham Adult Treatment Panel III (2001 and 2002) was used in 9 articles. The SCORE risk-estimation model developed for the European population was used eight times (see the other less-frequently used models in Table 1).

The study populations, eligibility, setting, and recruitment method are described in Supplementary Tables S2 and S8. In general, there was variation between the study populations regarding the age groups: seven models (10%) were developed for people with ages ranging from 30 to 74 years, and eight (11%) models were developed for the subjects with ages between 45 to 64 years, while the majority (*n* = 57, 79%) of the models used several different age groups (see Supplementary Table S2).

Most of the models (*n* = 53, 74%) targeted the general population (men and women), few models (*n* = 17, 23%) were developed for men, and only two (3%) models were available for women. Regarding the inclusion and exclusion criteria in most studies (*n* = 47, 71%), the researchers stated that participants with a history of coronary heart diseases (including a history of unstable angina or acute myocardial infarction, recorded ECG, stroke, heart attack), other diseases such as cancer (*n* = 6, 9%), diabetes mellitus (*n* = 10, 15%), or chronic medical conditions were excluded. Participants who were taking lipid-lowering medication or aspirin were also excluded from several studies. Additionally, few studies (*n* = 7, 5%) excluded participants because of race/ethnicity status, and one model had no information. In the modelling studies with genetic parameters, the investigators explicitly stated that they excluded study participants with no genotypic data (*n* = 11, 15%).

#### 6.1.2. Definition and Method for Measurement of Outcome

We observed a large variation in the definition of the population outcomes. The majority (*n* = 42, 58%) of the prognostic models defined CHD events as an incident of CHD with no categorization, while some models (*n* = 27, 38%) specified the definition of the outcome as (fatal or nonfatal) myocardial infarction, stable or unstable angina, percutaneous coronary revascularization or bypass grafting, or death due to CHD. Additionally, the international classification codes showed heterogeneity (*n* = 27, 38%) (see Supplemental Table S3). The definition of outcomes showed considerable heterogeneity; there were more than 20 different definitions for coronary heart disease outcomes. Other outcomes were identified, such as fatal/nonfatal CVD events (*n* = 1, 1%), and three models (*n* = 3, 4%) with no information (Supplemental Table S4).

#### 6.1.3. Time Span of Prognostic Models

The follow-up time period in the prognostic models reviewed ranged between 3–30 years, 4 models (6%) predicted the incidence of CHD for less than 5 years, 33 models (56%) predicted CHD outcomes for 5–10 years, a longer (>10–15 years) follow-up was described in 29 models (40%), and the length of follow-up was longer than 15 years in a few models (*n* = 6, 8%).

#### 6.1.4. The Candidate Predictors

**Figure 3:**
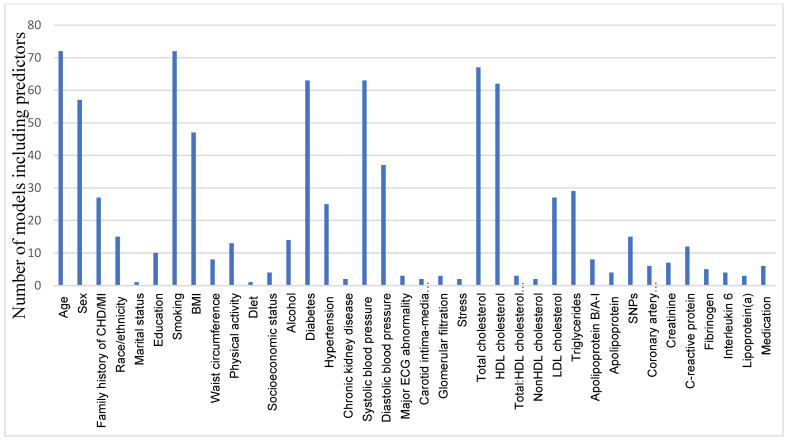
The main categories of predictors used in prediction models for CHD diseases. Several novel predictors were added to the Framingham model for predicting CHD events. Framingham predictors were used the most commonly (age, sex, smoking, SBP, TC, HDLC, and diabetes) compared to other predictors.

Figure 3 describes the set of predictors used in the prognostic models. In general, more than 237 different predictors were included. The major categories of the predictors used were conventional risk factors (number of predictors ranged between 7–20), genetic risk variables (ranged between 1–153 SNPs) and biomarker variables (ranged between 1–141). The categories of the predictors described were demographic characteristics, genetics, biomarkers, comorbidities, behavioural factors, physiological factors, metabolic syndromes, and reproductive factors (see Supplementary Figure S1). Age and smoking as predictors for CHD were used in all the studies, total cholesterol level was reported in 67 (93%) models, HDL cholesterol level was used in 62 (86%) models, diabetes mellitus and systolic blood pressure were used in 63 (87%) models, gender was included as a predictor in 57 (79%) models, 53 (73%) models used both male and female subjects, 17 (23%) models were specified for men, and 2 (3%) models were specified for women. Most of the models (*n* = 46, 63%) included a set of similar predictors, such as age, sex, smoking, total cholesterol, blood pressure, BMI, blood cholesterol/HDL cholesterol level, and diabetes mellitus. Other prognostic models included several different variables, such as hypertension (*n* = 25, 35%), family history of CHD and LDL cholesterol (*n* = 27, 38%), triglycerides (*n* = 29, 40%), genetic risk score (*n* = 17, 23%), C reactive protein (*n* = 12, 16%), apolipoprotein B (*n* = 8, 11%), and coronary artery calcification (*n* = 6, 8%). Treatment as a predictor for CHD was included in a few studies (*n* = 6, 8%), described as the use of antihypertensive/antidiabetic and lipid-lowering medications.

#### 6.1.5. Sample Size and Number of Outcomes

The number of participants used to develop and validate the prognostic modelling studies ranged between 112 and 268,315 (median 4651); almost half of the models (*n* = 50, 69%) recruited their participants from multiple centres, while (*n* = 16, 22%) recruited subjects from one centre, (*n* = 2, 3%) recruited from two centres, and 4 models (6%) did not describe the recruitment method. Regarding the study setting (primary or secondary health care centres), 45 models described that they selected the participants from primary health care centres, 4 (6%) models selected the participants from secondary health care centres, and 23 models described that they selected the participants from the communities by using a random selective sampling procedure. The number of outcomes that occurred during the follow-up period (CHD events) ranged between 56 and 203,666 (median 467), and two (3%) models did not report the number of outcomes.

#### 6.1.6. Missing Data

The number of participants with any missing value and whether the subjects were censored, or whether migration happened to the participant during the follow-up were described in 27 (37%) models. Methods for handling the missing data were described for several models. Seventeen models explained that they excluded the participants with missing data before starting the analysis, whereas four models reported that they used the imputation resampling technique, and only one reported that they repeated the measurement. Genetic modelling studies reported that they excluded the participant whether they missed information related to genetic data, ECG, and C reactive protein, blood pressure, total serum cholesterol, fasting serum glucose, smoking status, and body mass index.

#### 6.1.7. Modelling Method

Regarding the statistical analysis technique, 47 (65%) models used Cox proportional hazards to develop the prognostic model for CHD; logistic regression was used in 17 (24%) studies. Furthermore, conditional logistic regression and lifetime survival analysis were described in some models (*n* = 6, 8%) (Supplementary Table S5).

#### 6.1.8. Models’ Assumptions and Normality Distribution

Regarding the distributional assumptions about the residuals and whether the researchers selected the right predictors in their models, 18 models reported how they checked the assumption of the normality distribution using linear regression, seven models used Schoenfeld residuals to verify the proportional hazard assumption, and two models reported that they fit the models by Grambsch and Therneau (Supplementary Table S6).

The methods used for selection of the best predictors during multivariable modelling were a backward approach in 3 (4%) models, forward selection in 3 (4%) different models, Bayes information criterion (BIC) in 9 (13%) models, Akaike information criterion in 5 (7%) models, likelihood ratio test (LR) in 12 (17%) models, and Shrinkage or penalized estimation in 3 (4%) models (Supplementary Table S6).

#### 6.1.9. Predictive Performance of the Studies

The performance of a statistical prognostic model’s assessment showed considerable heterogeneity, and the discrimination measures of predictive performance were reported for 72 (100%) models (See Supplementary Table S9). Most of the models used the concordance index (Harrell’s C statistic) or area under the receiver operating characteristic curve (*n* = 57, 79%); seven (10%) models used the D statistic; and lifetime risks for CHD were reported in six (8%) models, and only one model described the log rank (Table 2). Calibration measures were reported in 29 (45%) different models, calibration slope and intercept were measured in 3 (4%) models, calibration plots were reported in 2 (3%) models, Hosmer–Lemeshow was used to assess the differences between the observed and expected rates in 20 (28%) models, and Grønnesby and Borgan was used to test the goodness of fit of 5 (7%) models (see Table 2). Classification measures were used to evaluate risk predictions in 54 (75%) models, sensitivity and specificity measures were used in 24 (33%) models, net reclassification improvement (NRI) was reported in 28 (39%) studies, integrated discrimination improvement (IDI) was used in 16 (22%) models to quantify how close prognostics were to the actual outcome, and clinical NRI was calculated to assess the improvement between the basic and extended models in 3 studies (4%). Regarding other methods, Kaplan–Meier survival curves were used in sixteen (22%) models. In total, 45 (63%) of the 72 models were developmental (internally validated), most often using a random split of the dataset (*n* = 18), bootstrapping (*n* = 21), or cross-validation (*n* = 5), and few models (*n* = 6) used multiple imputation to impute the missing values on all predictors.

## 7. Validation Modelling Studies

Validations of the models were performed in only 24 models: 10 (16%) models were developmental validation studies, and 14 models were external validation studies. However, the ten models had been developed and validated in the same research using different populations. Of these 10 models, 3 included genetic risk scores, while the other 14 were validated internally. Framingham models were the most common models used by researchers: Framingham developed by Wilson and D’Agostino in 1998 was reported in 33 models, Framingham of Adult Treatment Panel III 2002/2001 was described in 9 models, Framingham developed by Anderson in 1991 and Framingham developed by Kannel in 1979 were reported in 5 different studies. Our review identified other different models that were used to predict CHD development, such as SCORE (2003), which was described in seven models. PROCAM (developed by Assmann in 2002) was reported in three different studies. QRISK2 (2008) was used in two models, while 20 other studies did not specify which reference models were used (see Table 2).

There was considerable heterogeneity in the external validation modelling studies (specifically the developmental validation types) regarding the eligibility criteria used for the participants included. The different age groups were used for developing and validating the models. Most of the models measured the discrimination ability using the c-statistic only, and few models described the calibration measure (*n* = 5, 7%).

Our review identified that only two validation models compared the performance ability in different populations. The first model was a genetic risk modelling study based on the Framingham risk score, which measured the performance using discrimination, calibration, and reclassification in three different populations of the ARIC (Atherosclerosis Risk in Communities) study, Framingham Offspring Study, and Rotterdam Study (Netherlands) [46]. This work revealed conflicting results regarding model performance: the discrimination ability and reclassification showed significant improvement in the developed model but not in the validated models. The second study examined whether the model performance could be improved by adding coronary artery calcification as a marker predicting CHD risk [47].

The model compared three different populations of the Multi-Ethnic Study of Atherosclerosis, Heinz Nixdorf Recall Study, and Dallas Heart Study. These models measured the performance using discrimination and calibration, and the results of the study showed significant improvements in risk prediction by adding coronary artery calcification to conventional risk factors. Additionally, evidence of very good discrimination and calibration was provided.

## 8. Genetic Risk Prognostic Modelling Studies

The genetic risk modelling studies identified in this review were used to examine whether the inclusion of genetic factors improved the CHD risk prognosis in addition to conventional risk factors based on the Framingham score. Most of the models were developed in healthy populations in longitudinal cohort studies (*n* = 16), while one study used a nested case-control design.

Most of the genetic modelling studies originated in Europe (*n* = 11) and the United States (*n* = 5), and only one model was from Asia (China). The European modelling studies included four studies from the United Kingdom and one model each from Sweden, Denmark, Spain, Switzerland, the Netherlands, and Scotland. Most of the models (*n* = 11) were developed using Caucasian populations (white non-Hispanic).

The healthy participants of the genetic models were recruited from multiple centres (*n* = 12). The periods of recruitment ranged between 1987 and 2007; seven models recruited participants from 1987 to 2001, five models reported that the participants were recruited from 2003 to 2007, and two models provided no information. Regarding the age group, most of the models (*n* = 11) were developed for people aged 45–75 years, three models were developed for people aged 25–64 years, and two models had no information.

Most of the studies were developed using healthy Caucasian populations of both sexes with complete genetic information. The authors excluded all the participants with missing genotype data, individuals who had prevalent CHD or stroke, and individuals with no follow-up data. The number of participants ranged from 840 to 51,954, and the number of events ranged between 183 and 3217.

Genetic risk scores (GRSs) with various numbers of SNPs were included in the models reviewed. The total number of SNPs reported in the articles was 230. It ranged between 1–156 SNPs per article: four models utilized a relatively low number of SNPs (1–19), and thirteen models included a relatively high number of GRSs ranging between 38–156 SNPs. The most reported SNPs were rs17114036 (PLPP3) reported in 12 articles; rs1122608 (SMARCA4) and rs3184504 (SH2B3) reported in 7 articles; rs9818870 (MRAS), rs67258870 (DHRSX), and rs501120 (unknown) in 6 articles; and rs7692387 (GUCY1A1), rs12413409 (CNNM2), rs9515203 (COL4A2), rs11556924 (ZC3HC1), rs11206510 (unknown), rs273909 (SLC22A4, MIR3936HG), rs12190287 (TCF21), rs2048327 (SLC22A3), rs12526453 (PHACTR1), rs4252120 (PLG), rs2505083 (JCAD), rs974819 (unknown), and rs9982601 (unknown) in 5 articles. Other SNPs were less commonly used (see Supplementary Table S7). These SNPs were associated with well-known phenotypic traits or biomarkers that were associated with systolic blood pressure, total cholesterol, LDL-C, HDL-C, apolipoprotein-B, lipoprotein (a), plasma C reactive protein, health behavioural factor (smoking), and family history. Most of the selected SNPs (*n* = 9) were identified in genome-wide association studies and the CARDIoGRAMplusC4D (*n* = 4) study. One model described that they included SNPs based on a literature review.

Heterogeneity was observed in the genotyping technique used for analysis, which included Illumina MetaboChip in three different models, Affymetrix GeneChip (*n* = 2), custom-designed Affymetrix Axiom arrays and genome-wide arrays (*n* = 1), TaqMan technology in three models and MassARRAY (*n* = 1), and the other models had information reported.

Regarding the calculation of weighted GRS in the models reviewed, most (*n* = 12) of the models weighted the GRS by multiplying the participants’ allele score (1, 0, −1) by the SNP beta coefficient, while two models reported that they weighted the GRS by multiplying the number of risk alleles with the ‘combined beta’ of the CARDIoGRAMplusC4D meta-analysis and summing the products.

The GRS variables in the developed models were categorized as tertiles, quartiles, and quantiles. Most of the models *n* = 11 used the quartile as low GRS (quartile 1), intermediate (quartiles 2 and 3), and high (quartile 4) risk categories; tertiles were described in (*n* = 5) models such as low GRS (tertile 1), intermediate (tertile 2), and high (tertile 3) risk categories; only one model used quantiles including low GRS (quintile 1), intermediate GRS (quintiles 2 to 4), and high GRS (quintile 5).

The follow-up time of the models ranged from 5 to 19.4 years. Seven models predicted CHD outcomes in >10–15 years, six models described the follow-up time as 5–10 years, two models described a follow-up period of less than 5 years, and another two models reported that the length of follow-up was longer than 15 years. Most of the models (*n* = 12) reported that they collected the data using questionnaires, physical examinations, and laboratory diagnostics. Genotype imputation was performed in five models, and bootstrapping was described for *n* = 5 models.

Most of the studies (*n* = 6) estimated the ten years of CHD risk using the Framingham risk function described by Wilson et al., the Framingham Adult Treatment Panel III was used in (*n* = 3) models, and other models did not specify which Framingham model was used.

The performance of genetic modelling studies was reported in (*n* = 16) models, discrimination measures using the concordance index (Harrell’s C statistic) or area under the receiver operating characteristic curve were reported in (*n* = 13) models, calibration measures were reported in (*n* = 10) models, Hosmer–Lemeshow goodness of fit was reported in (*n* = 5) models, and Grønnesby and Borgan was reported in (*n* = 2) models. Classification measures were reported in (*n* = 10) models, and calibration measures using the net reclassification improvement (NRI) were reported in (*n* = 10) models.

## 9. Discussion

This review shows that numerous prognostic models have been developed for CHD risk prediction in the general healthy population, and the predictors are based mainly on Framingham models, but there is still no consensus about the best model(s). Our review adds some new and structured knowledge about CHD prognostic modelling studies by including models with genetic risk components in addition to traditional models.

Although the number of publications on clinical prognostic modelling studies combining multiple predictors for CHD has increased in the medical literature [17], the translation of the knowledge gained is missing, and limited evidence is available on the application of genomic results in health and public health practice [110]; consequently, the trend of CHD mortality and morbidity is still increasing consistently over the years, especially in low- and middle-income countries [111].

We hypothesized that adding the genetic risk score to conventional risk-factor-based models would improve the predictive ability of these models regarding CHD events in the general population. However, we identified that most of the genetic modelling studies had not yet been externally validated in different populations, with the exception of a distinguished model [52]. Most of the genetic studies have shown improvement in performance (discrimination and calibration ability) without further external validation [40,41,42,43,44,46,47,48,49,50,51,52]. Demonstrating the good performance of the models developed is not sufficient without confirming improvements in different populations for the generalizability of the models.

Healthcare providers and policymakers believe that the reduction in deaths and disability due to CHD will be more influenced by prevention, not treatment, and this requires robust reduction of risk factors through the accurate estimate of the population at risk [112]. Clinicians are also willing to find suitable guides for quick decisions in everyday medical practice to improve patient outcomes as much as possible [113]. One recommended solution would be the validation of the existing models instead of building several new models; however, the translation and dissemination of results into practice is challenging, and no prognostic application is ready for implementation in routine daily practice.

Our review identified variation regarding the geographical location of models: most models were developed and validated in the United States of America, Canada, and European populations. The WHO stated that more than three-quarters of all CHD deaths occur in low-income and middle-income countries [111]. Our study confirmed that no prognostic modelling studies for CHD originated in developing countries, e.g., a prognostic model for people from Africa had not yet been developed when we conducted the search for this review. Only a few studies originated from Asia (China, Turkey, Thailand, Japan, and Korea).

Most participants were recruited from primary health care and community settings, but the selection process was incompletely reported. Most of the studies did not describe which sampling technique was used, and few studies described random method selection of the participants. Sampling techniques are an important method for representative target populations; researchers should be more accurate in this era to avoid selection bias and to provide clinicians with valid information. Few studies reported consecutive participant selection, but in some other previous studies, participants were selected nonconsecutively and thus increased the risk of bias due to selective sampling [114].

Although age is the most common risk factor considered in CHD risk prediction and affects the two sexes for developing CHD, there is great variation between the developed and validated models in terms of the participant age groups; the validation studies were performed in people outside the age range of the developmental studies, and most of the developed models predicted the risk in the elderly population compared with the validated models, which may affect the number of CHD outcomes and may influence the model performance [115]. Researchers should specify the age group by sex because females have a lower risk of developing CHD than males [116].

Although disparities of CHD risk depend on factors such as age, gender, and geographical location of populations, only a few researchers are focusing on females and middle- and low-income countries possibly due to data availability, population needs, or priorities. Priorities for studies in developing countries (Africa and Asia) are different from Europe or America. Developing regions of the world must focus on the leading causes of death such as malaria, HIV and AIDS, dengue, and tuberculosis [117]. Developmental validation studies should compare diseases in the same age group in both of the models developed and validated.

The method of outcome determination should be accurate to provide proper patient risk stratification and to support personal clinical decision making with the goal of improving patient outcomes and quality of care [118]. Incorrect outcome assessment and measurement of predictors may inflate the predictive accuracy of the predictors and that of the final prognostic model [15]. With respect to CHD definitions reported in the models reviewed, most models defined the incidence of coronary heart disease as fatal or nonfatal myocardial infarction, and over 80 different definitions for the disease outcomes were identified. In addition, most outcomes were not fully defined, and the International Classification of Diseases (ICD) codes for CHD were described in only a few models. Different outcome definitions and measurement methods may lead to differences in study results and are a source of heterogeneity across studies, and thus risk of bias [15]. Overall, heterogeneity of definitions of CHD outcome identified in this review may affect the discrimination ability of the models (overestimate/underestimate): models fail to discriminate between case and non-case subjects and thus may influence the predictive accuracy of the final prognostic model [119]. A standard consistent definition of CHD and outcome will increase transparency in reporting the predicted outcomes and may improve the quality of research.

Several candidate predictors were reported in the models identified. Most models had the same common predictors, such as age, sex, smoking, blood pressure, and total cholesterol levels. Many novel predictors, such as genetic risk scores, biomarkers (coronary artery classification and C-reactive protein), and others (e.g., creatinine, fibrinogen, and interleukin 6) have been described in a few models. Most of the novel and newly developed models show good performance in predicting CHD, but the strategies of how the predictors were selected (backward or forward stepwise regression) and which approaches were used (plot or multiple linear regression) are still questionable in most studies reviewed (Supplementary Figure S2).

Regarding the models’ performance, most of the models measured discrimination ability with less commonly used calibration and classification measures. Therefore, the performance of these models has not been fully examined, and the discrimination alone can be insensitive and less useful in evaluating risk prediction of future events. It will be valuable if it is used for comparing the fit of predictive models using the calibration statistic and reclassification improvement [120]. It is essential to assess the goodness of fit and to validate the model to ensure predictive performance. The use of good strategies for model selection in addition to adequate performance and goodness-of-fit measures is needed in developing accurate predictions [121]. It is important to ensure that the model is well-calibrated if the prognostic value is close to the true value of disease outcomes. Model calibration was assessed via Hosmer–Lemeshow goodness of fit with other measures, such as adjusted R square, cross-validation, and Akaike’s and the Bayesian information criterion for small numbers of predictors [122]. If there are a large number of predictors, then forward stepwise regression can be used [121].

Our review assessed prognostic modelling studies in community-based settings, aiming to summarize the available evidence about the optimal model in predicting CHD events in healthy populations and to explore whether the inclusion of the genetic risk score in conventional risk factors improves the ability of these models. MESA risk score with coronary artery calcification, which is described by McClelland et al. (2015) seems to be an optimal model for predicting CHD risk in the general population [91]. This model is a conventional modelling study including age, gender, CAC, ethnicity, DM, smoking, family history of heart attack, TC, HDLC, SBP, and treatment (hypertension and lipid lowering) predictors. We would recommend this model to be used in low-income counties countries (it is less expensive compared to GRS models). Genetic risk score (unweighted and weighted) in the model of Brautbar et al. (2012) might help in predicting CHD risk when integrated to Framingham [52].

These findings can help clinicians and decision makers improve the quality of interventions and improve the health of the population at risk. Future validation studies for genetic modelling studies are needed to ensure the quality and transparency of the developed model. Methodological assessment of genetic models is required. Most of the genetic risk scores incorporated into the conventional risk factors improved the discrimination and reclassification ability in the derivation models. Most genetic modelling studies were developed using only Caucasian populations; thus, the generalizability of the existing prognostic models is questionable. Genetic modelling studies might be used to target the prevention of CHD if the individual’s genetic risk is comprehensively evaluated. An accurate assessment of an individual’s risk is fundamental to future efforts in personalized medicine for the primary prevention and proper management of CHD.

Future validation studies should include genetic application in different geographic locations, and fully independent validation by independent investigators using alternative measurements of these risk factors in different population settings may improve the prognosis of the disease.

Incomplete reporting of information in both conventional and genetic modelling studies was observed regarding the following methodologies: sampling technique, subject selection criteria, categories and blinding, genetic information (selection, coding), construction of the final models, classification measures, duration of follow-up, and missing values of the participants and technique used for handling this issue (bootstrapping, cross validation, and resampling method). Therefore, simply excluding the participants with missing values from the analysis reduces the effective sample size and may also lead to inaccurate estimates of the predictor outcome associations and the predictive performance of the final model [13]. The performance of a predictive model is overestimated when simply determined on the sample of subjects that was used to construct the model, and statistical techniques such as shrinkage and bootstrapping are available to attempt to reduce over optimism at the model-building stage [17,121]. Comprehensive and valid information on conventional and genetic models is needed. Researchers should enhance the quality of their reports by describing and highlighting this important information.

To ensure the generalizability of the prognostic model and the ability of the model to predict CHD in populations with different characteristics, an external validation study is needed to evaluate the model performance and to avoid overfitting in prognostic modelling studies. The shrinkage and penalization method should also be applied to reduce overfitting by readjusting the regression coefficients [13,14].

Our review reveals that only one conventional model was considered a good prognostic model for CHD in the general population (decision analysis) and applicable for use in clinical practice (classification marker for CHD risk prognosis) [91]. One genetic modelling study (Brautbar et al., 2012), was externally validated in three different populations and performed decision analysis but had limitations regarding classification improvement in the comparator model [52].

For conventional and genetic modelling studies, the identified previous models might be considered a good and optimal prognostic for CHD risk in the general population (decision analysis) and applicable for use in clinical practice (classification marker for CHD risk prognosis). However, the model of Brautbar et al. (2012) was externally validated in three different populations and performed decision analysis, but this model had limitations regarding the discrimination and classification improvement that occurred in the developmental group without significance improvement in the comparator groups [52].

There are several reasons why the performance of a prognostic model needs to be evaluated before its results can be used; most of the models fail to satisfy certain statistical notions of correctness (statistically invalid), fail to be useful in a clinical setting, or have invalid prognostic information. Furthermore, the same model might fail according to one clinical criterion and pass according to another. There were two definitions of the validation prognostic modelling study. First, a statistically validated model passes all appropriate statistical checks, including the goodness of fit on the original data set and unbiased prognosis on a new data set. Second, a clinically validated model performs satisfactorily on a new data set according to context-dependent statistical criteria [123].

## 10. Conclusions

Although the heritability of CHD is recognized and confirmed, the understanding of genetic architecture of CHD is still uncertain, and most of the SNPs’ functions for CHD risk prediction are unknown or less-known. Our review assessed prognostic modelling studies in community-based settings, aiming to summarize the evidence about the optimal model in predicting CHD events in healthy populations and to explore whether the inclusion of the genetic risk score alongside conventional risk factors improves the predictive ability of these models. MESA risk score with coronary artery calcification (CAC) which is described by McClelland et al. (2015) seems to be an optimal model for predicting CHD risk in the general population (this model includes age, gender, CAC, ethnicity, DM, smoking, family history of heart attack, TC, HDLC, SBP, and treatment (hypertension and lipid lowering) predictors). We would recommend this model for use in low-income countries (it is less expensive compared to GRS-based models). The genetic risk score (unweighted and weighted) from the model of Brautbar et al. (2012) was very powerful in predicting CHD risk when it was integrated into Framingham conventional factors. Our findings can help clinicians and decision makers to improve the quality of interventions and improve the health of the population at risk. Future validation of genetic modelling studies is needed to ensure the quality and transparency of the developed models. Methodological assessment of genetic models is required. Most of the genetic risk scores incorporated into the conventional risk-factor-based models improved the discrimination and reclassification ability. Most genetic modelling studies were developed using only Caucasian populations, so the generalizability of the existing prognostic models is still questionable. Genetic modelling studies might be used to target the primary prevention of CHD if the individual’s genetic risk is comprehensively evaluated. An accurate assessment of an individual’s risk is fundamental to future efforts in personalized medicine.

## 11. Strengths and Limitations of This Study

In this study, multiple databases were searched (Embase, PubMed, Cochrane, web of science, and Scopus), applying a human filter only. Data extraction was performed by one reviewer and then checked by another one, and characteristics of the individual studies were provided. Quality assessment was performed in duplicate using CHARM, and GRIPS statements and the data collected were not suitable for conducting meta-analysis because of the heterogeneity and the huge number of different predictors identified.

## Figures and Tables

**Table 1:** List of the models that were developed and validated for predicting coronary heart diseases in the general population.

No	Name of the Models	Frequency of the Models		
		Developmental	Validation	Total
1	Framingham–Wilson–D’Agostino, 1998	11	9	20
2	SCORE 2003	5	2	7
3	Framingham–ATP III, 2002	6	0	6
4	Framingham–Anderson, 1991	2	3	5
5	Framingham–Kannel, 1979	2	3	5
6	Framingham–Wilson, 1998	4	1	5
7	Framingham–ATP III, 2001	1	2	3
8	PROCAM–Assmann, 2002	3	1	4
9	Framingham–D’Agostino, 2008	3	0	3
10	QRISK2–Hippisley-Cox, 2008	0	2	2
11	PROCAM–Assmann, 2007	0	1	1
12	Framingham–Splansky, 2007	0	1	1
13	Framingham–Kannel, 1959	0	1	1
14	Framingham–Kannel, 1986	1	0	1
15	Framingham–Polak, 2011	1	0	1
16	Framingham–Wilson, 1991	1	0	1
17	Framingham–Wang, 2006	1	0	1
18	Framingham–Wilson, 2005	0	1	1
19	Framingham–Ridker, 2002	1	0	1
20	Framingham–Franklin	1	1	2
21	Framingham–ARIC, 2003	1	0	1
22	Framingham–Rotterdam	2	0	2
23	Framingham–MESA, 2002	1	1	2
24	Framingham–Lee, 2016	1	0	1
	Framingham (not specified)	19	4	23
Total		67	33	100

**Table 2:** The performance measures reported for the developed and validated models.

No	Discrimination Measures	Developmental	Validation	Total
1	C statistic/AUC	54	9	63
2	D statistic	2	1	3
3	Log rank	0	1	1
4	Lifetime risks for CHD	3	2	5
Calibration measures:				
5	Calibration slope and intercept	0	3	3
6	Calibration plot	0	2	2
7	Hosmer–Lemeshow test	11	9	20
8	Grønnesby–Borgan χ^2^ test	4	1	5
Classification measures:				
9	Sensitivity, specificity	14	10	24
	Predictive value	5	2	7
10	Net reclassification improvement (NRI)	19	9	28
11	Integrated discrimination improvement (IDI)	10	6	16
12	Clinical NRI	0	3	3
Others:				
13	*R* ^2^	2	0	2
14	Kaplan–Meier estimates	11	5	16
15	Bootstrap resampling	16	5	21
16	Cross-validation	2	2	4

## Data Availability

Not applicable.

## Supplementary Materials

The following supporting information can be downloaded at: https://www.mdpi.com/article/10.3390/jcdd9090295/s1, Table S1. Describes the list of full-text articles identified and the reason of exclusion during the investigation of eligibility. Table S2: Description of the study populations, settings, locations, periods of recruitment, length of follow-up and method of data collection of the reviewed models. Table S3: coronary heart diseases definitions as described in the models reviewed. Table S4: Outcome’s categories of CHD used for models reviewed. Table S5: Modelling method used to develop the prediction models. Table S6: Method for selection of predictors in models included Table S7. SNPs included in the genetics modelling studies. Table S8. PICOTS elements of the articles reviewed. Table S9. Discrimination, calibration, and risk classification as described in the reviewed models Figure S1. Categories of predictors that reported in the models included in the review. Figure S2: The steps of developing a novel model Figure S3: Types of Prediction Modelling Studies identified Figure S4: The conventional and genetics risk models included.

## Abbreviations

ATP	Adult Treatment Panel
BMI	Body Mass Index
WC	Waist Circumference
CHD	Coronary Heart Disease
CRFs	Conventional Risk Factors
CVD	Cardiovascular Diseases
DALYs	Disability-Adjusted Life Years
DM	Diabetes Mellitus
GRS	Genetic Risk Score
HDL	High-Density Lipoprotein
HTN	Hypertension
ICD	International Classification of the Diseases
IDI	Integrated Discrimination Improvement
LDL	Low-Density Lipoprotein
MI	Myocardial Infarction
NRI	Net Reclassification Improvement
SNPs	Single-Nucleotide Polymorphisms
WHO	World Health Organization
