# Supplementary material for: Prognostic Modelling Studies of Coronary Heart Disease—A Systematic Review of Conventional and Genetic Risk Factor Studies
Source: J Cardiovasc Dev Dis. 2022 Sep 5;9(9):295. doi: 10.3390/jcdd9090295 (PMC9505820; doi:10.3390/jcdd9090295)
Supplement: Supplementary file 1 [file jcdd-09-00295-s001.zip › jcdd-1809380-supplementary.pdf]

Supplementary material for the manuscript entitled

Prognostic Modelling Studies of Coronary Heart Disease—A Systematic Review of Conventional and Genetic Risk Factor Studies

Nayla Nasr [1,2] Beáta Soltész [3], János Sándor [2], Róza Adány [1,2] and Szilvia Fiatal [2,*]

[1] Doctoral School of Health Sciences, University of Debrecen, 4032 Debrecen, Hungary
[2] Department of Public Health and Epidemiology, Faculty of Medicine, University of Debrecen, 4032 Debrecen, Hungary
[3] Faculty of Public Health, University of Debrecen, 4032 Debrecen, Hungary
* Correspondence: fiatal.szilvia@med.unideb.hu

Details of the selection process:

According to our objective we selected articles describing the prognostic modelling studies of CHD in the general population (subjects are free from CHD). Cohort studies (n= 66) and nested case control studies (n= 6) were included in the systematic review because the prospective studies allow the optimal documentation of predictors and outcomes. Case-control studies are not suitable according to the recommendations, therefore were excluded (Moons et.al, 2014).

The items of PICO framework:

P: People free of coronary heart disease
I: Developmental prediction models
C: Validation prediction models
O: Incidence of CHD within a specified time interval.

Questions of interest:

What is the optimal model or how good is a model in predicting CHD risk?
Which biomarkers/genetic markers should be incorporated in the risk prediction model beside the conventional factors?
Will the genetic information improve risk prediction?

Hypothesis:

Adding the genetic risk score (GRS) to the conventional risk factor (TRF) based models would improve the ability of these models to precisely predict CHD events.

Data Extraction:

The CHARMS checklist and GRIPS guideline were used

Study designs included:

Cohort studies nested case control and case-cohort.

Modelling of interest:

Three types of predictive modelling studies included: 1. Developmental, 2. Validation modelling and 3. Developmental validation studies

Population Description:

Describe the population selection criteria (inclusion and exclusion), race/ethnicity, geographical region, sample collection technique

The follow-up and duration in the prediction modelling study:

Time interval is important to see whether the participants developed CHD. The duration of follow-up of prediction modelling studies started from zero-time to the end period of follow up that specified in the study Follow-up and duration involved in prediction models should be described accurately in the study. Between the zero-time and the end of the follow-up researcher should do at least one more datum collection for accurate result (Moons et.al, 2015).

Statistical analysis for prediction modelling studies:

Logistic regression and proportional hazard regression analyses are required to describe the effect of the categorical predictors.

Model performance:

Models' performance can be assessed usually by using different method which included discrimination, calibration and reclassification measures. Discrimination measured by area under the receiver operating characteristic ROC curve (AUC), or concordance (or c) statistic; calibration measured by calibration slope, survival analysis (Hosmer-Lemeshow goodness-of-fit test), Grønnesby and Borgan test. Reclassification is measured by net reclassification improvement (NRI). The net reclassification index is a measure for evaluating the improvement in prediction

performance gained by adding a marker to a set of baseline predictors.

Risk categories in models included:

Conventional models based on Framingham risk score categories described as: 10% (low), 10-20% (intermediate), 20% or more is (high) risk of CHD at 10 years. While genetic risk prediction modelling studies described the weighted risk categories as: 0-5% (low), >5-≤10% (intermediate-low), >10-≤20% (intermediate-high), >20% (high) it used.

Table S1. Describes the list of full-text articles identified and the reason of exclusion during the investigation of eligibility.

No	Study design	Identified	Included	Not included	Exclusion reason
1	Longitudinal (Cohort)	346	66	280	Suspected patient/CVD and stroke
2	Case control	73	0	73	Potential bias
3	Nested case control	7	6	1	Intervention
4	Cross sectional	21	0	21	Diagnostic models
5	Case cohort study	5	0	5	Poor quality (No follow up).
6	Case report	2	0	2	Short follow up
7	Blinded comparison	1	0	1	Poor quality
8	Pooled analysis	9	0	9	No value of use it
9	No information	12	0	12	Poor quality
Total		476	72	404	

Prediction models identified:

Regarding to our objectives and PICO items work 72 articles described the prognostic models of CHD in healthy population is eligible for reviewing: which included 66 cohort study design and 6 nested case control, other models were excluded.

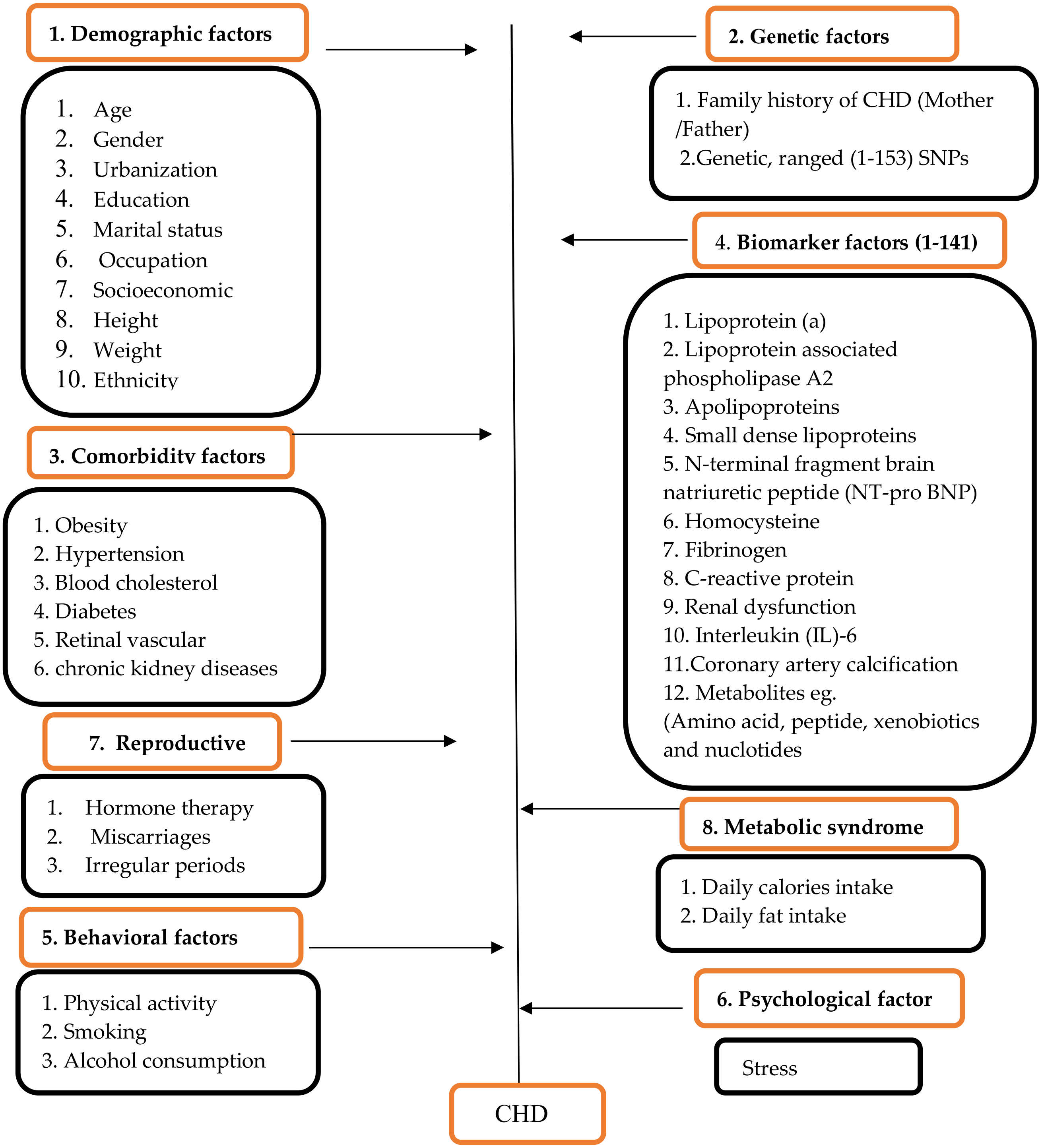

Figure S1. Categories of predictors that reported in the models included in the review

The predictors of CHD were reported in the models reviewed. They are categorised into different subgroups such as demographic, genetic, biomarker, comorbidity, behavioral, psychological, reproductive risk factors.

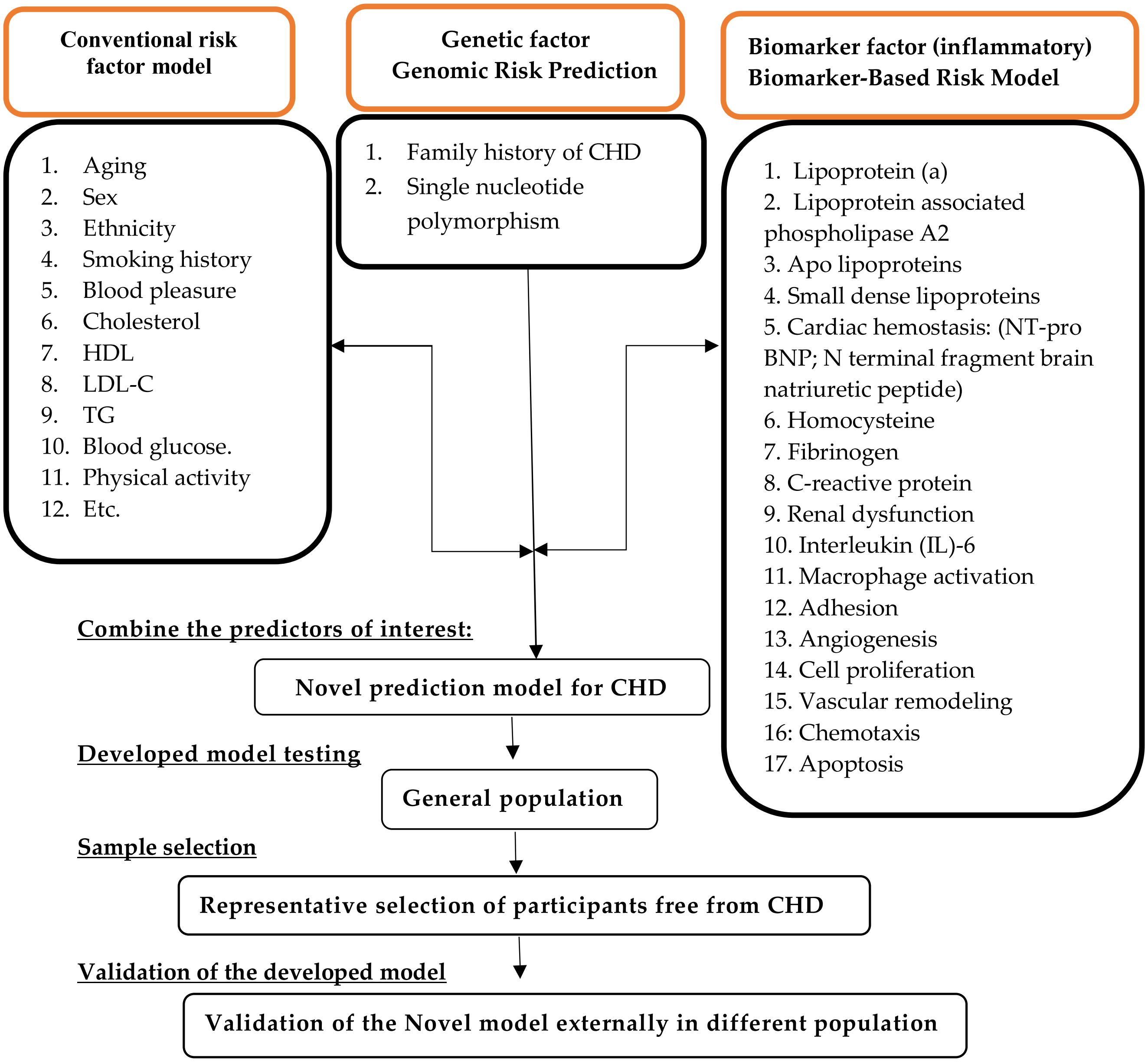

Figure S2: The steps of developing a novel model

The steps of developing novel model which includes: combining the predictors of interest, selecting the predictors to develop novel model for specific population/country, then developed model should be validated in other population/countries, assessing whether this model is optimal (valid and accurate) by measuring the performance (improvement in the discrimination, calibration and reclassification); then it can be used in clinical setting for CHD risk prediction in general population (generalizability).

Table S2: Description of the study populations, settings, locations, periods of recruitment, length of follow-up and method of data collection of the reviewed models.

No	Populations
1	The GERA cohort study of Genetic Epidemiology Resource in Adult Health and Aging, adult (men & women) free of CHD at baseline (2007-2008) were selected randomly by including all racial and ethnic minority participants from Kaiser Permanente of Northern California, 51954 participated, aged >30-<74, White non-Hispanic followed for a maximum of 5.9. All participants responded to a self-administered questionnaire that included information on medical history, ancestry, health behaviors.[1]
2	The MORGAM case-cohort study of (4818) healthy men at baseline, white Caucasian were selected randomly from nine prospective European cohorts, aged 25-64 years, over a median18 years follow up, participants used to examine the associations between CHD and risk scores based on genetic variants representing 13 genomic regions, baseline examination of this cohort took place in the year 1997.[2]
3	The second Northwick Park heart study (NPHSII) is cohort of 2742 white Caucasian, middle-aged men (50-64 years), recruited (2005) from nine United Kingdom general practices, questionnaire used to assess Family history of CHD. Participants with genotype data included 15 years followed for incident of CHD.[3]
4	2057 participants of the second Northwick Park heart study (NPHSII), middle-aged men (50-64 years), Caucasian men with complete trait data (and with full genotype data) included in this study, 10.8 years follow up, recruited in 2005.[4]
5	Two prospective studies compared; QRISK2 and NPHSII, the QRISK2 was used to assess CHD risk using conventional risk factors (CRFs), while Northwick Park Heart Study (NPHSII) used to assess the performance of a 19 single nucleotide polymorphism (SNP) gene score (GS) for CHD. NPHSII study included 2775 healthy UK men, aged (50-64) were recruited from nine general practices, 13.5 years' follow-up.[5]
6	The CoLaus study is an ongoing prospective survey investigating the biological and genetic determinants of cardiovascular disease in the population of Lausanne, Switzerland, (4283) Participants, aged 35-75 years, over median follow-up time of 5.6 years, the study relies on personal interview, physical examination and laboratory testing, the baseline investigation was conducted in 2003-2006 and a first follow-up in 2009-2012.[6]
7	Three different prospective studies were compared (ARIC, Rotterdam and Framingham Offspring). In total, 8542 of ARIC participants, aged 45-64 years, non-Hispanic whites followed for a maximum of 18 years, 2068 Rotterdam; all inhabitants of Ommoord, a district of Rotterdam in the Netherlands, aged under 65 years information from baseline (1990-1993) until January 1, 2007, and 2339 participants of Framingham Offspring Studies free of CHD at baseline and had genetic data, followed from study entry at exam 1 (1971-1975) for the first occurrence of CHD (incident CHD), data collected using questionnaire used and a clinical examination.[7]

8	Chin-Shan Community Cardiovascular Cohort Study of 3568 (women &men), homogeneous recruited from Chinese ethnicity, living in the Chin-Shan township, aged ≥35 years, with blood lipid data and free from CVD, 13.6 years' follow-up, the baseline investigation was conducted in 1990-2005.[8]
9	EPIC-Norfolk is a prospective cohort study in which men and women, 10295 participants, aged 40-79 years were recruited in 1993-1998 from general practices in the Norfolk region, England, 8.5 years' follow-up.[9]
10	This study is based upon 5191 men who at enrolment, Employees were recruited between 1970 -1973 from 27 workplaces in the west of Scotland, aged 35-64, 15 years' follow-up.[10]
11	A total of 5521 participants of Suita study, a Japanese urban population women and men, randomly selected, aged 30-79 years, free of CHD at baseline in 1989-2004, compared with original Framingham Score over median follow-up time of 11.8 years.[11]
12	3322 participants of The Framingham Offspring Study, residents of the city of Framingham, Massachusetts, (Men & Women), Participants who attended the fourth examination cycle (1987-1991), underwent a routine medical history, a physical examination that included blood pressure measurement and anthropometry and blood sampling were included, aged 30-74 years, 15 years follow-up.[12]
13	The study population was derived from the Multi-Ethnic Study of Atherosclerosis population (MESA), (4679) women and men, Caucasian, African-American, Hispanic, or Chinese-American participants, aged 45 to 84 years and free of clinically apparent CVD were recruited between 2000-2002, from (6) U.S. communities, 12.5 years' follow-up.[13]
14	A total of 13145 participants of Atherosclerosis Risk in Communities (ARIC), cohort study from US, aged 45-64 years, Caucasian non-Hispanic whites followed for a maximum of 15.1 years, recruitment year 1987-1989, who were followed for the development of clinical CHD.[14]
15	3971 participants of Cardiovascular Health Study (CHS), Boston men and women, blacks& white Invited to participate, selected randomly, aged ≥ 65 years were recruited between 1989-1990 or 1992-1993, 10 years' follow-up.[15]
16	The Second Northwick Park Heart Study (NPHS-II), 3052 participants, healthy UK men aged 50-64 years, Caucasian, followed for a median of 10.8 years for CHD events, the study compared the predictive value of the PROCAM and Framingham risk algorithms in (NPHS-II) population, recruited in 2004.[16]
17	The Normative Aging Study is a prospective study of aging established by the Veterans Administration in 1961, 1393 white males from a single geographic location (Boston, Mass), aged 30-74, 10 years' follow-up.[17]
18	Korean Heart Study (KHS) population, 268315 participants (women & men), selected non-random, who had voluntarily undergone private health examinations in 18 centers located in the capital and six provinces in South Korea between 1996 -2004, aged 70-79 years, 11.6 years' follow-up.[18]
19	The Dutch Cardiovascular Registry Maastricht (CAREMA) study population of Netherlands, 21148 participants (women& men), selected randomly from the Maastricht region, were recruited in 1987-1997, aged 20-59 years, 10.9 years follow-up.[19]
20	A total of 5101 from Tehran Lipid and Glucose Study, a prospective population-based study conducted on a representative sample of district-13 of Tehran, (Iranian urban population men and women), aged ≥30, free of CHD at baseline, were recruited in 1999 -2001, 9.3 years' follow-up.[20]

21	Participants were part of the Health, Aging, and Body Composition Study (Health ABC Study), a population-based cohort study of 2192 community-dwelling men and women, selected randomly (white& black) aged 70-79 years recruited in 1997-1998, over median follow-up time of 8 years.[21]
22	630 participants of active-duty US Army personnel-stationed within the National Capital Area of the Walter Reed Health Care System were included, aged 39-45 years, free of CHD, 5 years follow-up, recruited in 1998-1999.[22]
23	25663 participants of the European Prospective Investigation into Cancer and Nutrition (EPIC)-Norfolk study, resident in Norfolk, UK, participants who completed a baseline questionnaire survey and attended a clinic visit selected randomly to participate in a nested case-control study (men & women), aged 45-79 years, recruited in 1993-1997, 6 years' follow-up.[23]
24	72982 participants of The Women's Health Initiative (WHI) study in the prospective cohort analysis: median follow-up was 12 years, free of CHD, recruitment in 1991-1993.[24]
25	A total of 5899 participants of Rotterdam cohort Study, from Ommoord, a district of Rotterdam in The Netherlands, aged 55 years old or over, invited to participate. Baseline examination lasted from 1990 -1993, 12.8years Follow-up.[25]
26	The ARIC study is a prospective investigation of atherosclerosis, 5533 and 7301 individuals (men and women) selected from four U.S. communities, aged 45-64 years, 3 years Follow-up prior to the baseline, recruitment (1986-1989).[26]
27	The ARIC is prospective investigation of atherosclerosis its clinical sequelae, included 15792 US, aged 45-64 years, recruitment in (1986-1989), individuals were genotyped for 116 single nucleotide polymorphisms associated with CHD in multiple case-control studies, 1452 CHD cases defined as participants with either definite or probable myocardial infarction and 13,907 participants were followed for incident CHD for a median of 13 years between the baseline examination and December 31, 2001.[27]
28	A total of 15792 participants of the Atherosclerosis Risk in Communities (ARIC), case-control studies were included to assess the association of 19 novel risk markers with incident CHD, aged 45-64 years, sampled from four U.S. communities, in (1987-1989).[28]
29	A 1196 participants study (men &women) were recruited from the South Bay Heart Watch prospective study designed in 1990-1992, they were received mail letters invitation to participate in this study, subject who agreed to return for testing and be followed up for an additional 3 years included, the mean aged of the participants was 66 years, 3.5 years Follow-up.[29]
30	23595 participants of the Malmo¨diet and Cancer study (prospective, population-based study), a residents of Malmo, Sweden, men and women invited to participate, during a median follow-up of 14.4 years, recruitment in 1991-1996, aged 46 -73 for Men and 45-73 for women.[30]
31	A total of 2536 male employees from the Electricity Generating Authority of Thailand were participated in this cohort study, 17 years of follow-up, aged 35-59 years at baseline were included in the study.[31]
32	The Edinburgh Artery Study (EAS) is a prospective study from Edinburgh, Scotland, in general 840 men and women randomly selected to examine whether a panel of SNPs, systematically selected from genome-wide association studies (GWAS), could improve

	risk prediction of coronary heart disease (CHD), participants aged 54-75, 15 years of follow-up. Clinical examinations were held during 1987-1988, complete follow-up was available until June 2003.[32]
33	6216 participants (men and women) of the Framingham Heart Study (Massachusetts, USA) were included, All subjects were examined from 1971-1996 who were free of CHD, they stratified into age and gender-specific tertiles of Framingham risk score, and lifetime risk for CHD was estimate, aged 40-94, 10 years Follow-up.[33]
34	PRIME is Prospective Epidemiological Study of Myocardial Infarction, it was recruited in centers in Belfast (Northern Ireland) and Lille, Strasbourg, and Toulouse (France) and a Coordinating Centre in Paris, this study was compared with Framingham and PROCAM risk function to assess whether the Framingham and PROCAM risk functions were applicable to men in Belfast and France. In general, the PRIME study is comprised men recruited in Belfast (2399) and France (7359) who were aged 50 -59 years, free of CHD at baseline (1991-1993) and followed over 5 years for CHD events. Two validation study of the original Framingham & Framingham Offspring cohort consisted of 2489 men aged 30-74 years, who were free of any cardiovascular disease at the time of their examination from 1971-1974 within 10 years and PROCAM risk function study estimates the risk function of CHD and myocardial infraction within 10 years, it was developed from a sample of men, It consisted of 5389 men aged 35-65 years who were free of any cardiovascular disease at baseline between 1979 and 1985.[34]
35	2193 Participants (men & women) of the part of the Health, Aging, and Body Composition Study (Health ABC Study) were included, a population-based cohort of community-dwelling, aged 70-79 during the study enrollment period in 1997-1998. Participants were identified from a random sample of white and all black, 8 years Follow-up.[35]
36	A total of 9155 Participants (men & women), without diabetes selected from the Atherosclerosis Risk in Communities (ARIC), 4 US communities, participants included to investigate whether quantitative retinal vascular caliber are associated with an increased risk of incident coronary heart disease, aged 45-64 years, enrollment period in 1987-1989, a mean of 8.8 years of follow up.[36]
37	A total of 2259 subjects (men & women) from the Turkish middle-aged adults population (TARF study) participated in this study, the study is based on a longitudinal follow-up of survey conducted initially in 1990 in all geographic regions of Turkey, they were selected randomly, aged 35-84, during 1990-1995, 5 years of follow up.[37]
38	Participants of Framingham cohort Studies consisted of 2489 men and 2856 women 30-74 years old at baseline with 12 years of follow-up in 1971-1974. Participants attended either the 11th examination of the original Framingham cohort or the initial examination of the Framingham Offspring Study included.[38]
39	Framingham risk score were calculated for participants in the ARIC cohort, 3901 men, 5406 women were participated in this study from 4 US communities, aged 45-64 years old at entry (1986-1989 through 2001),at baseline (visit 3) and (3 - 6) years before.[39]
40	This study is based on a cohort of 970 men who were 34-64 years of age and free of CHD, other cardiovascular disease, and diabetes while participating in the second examination of the Helsinki Policemen Study in 1971-1972, 22 years of follow up.[40]
41	Data from two population studies of Glostrup (n=4757) and Framingham Heart studies (n=2562) were included to examine three different level of cross validation of risk score of CHD, participants (men & women), aged 30-70, free of myocardial infraction disease, 10 years follow-up.[41]

42	The HUNT studies were carried out in the Nord-Trøndelag County in Norway in three waves, HUNT1(1984-1986), HUNT2 (1995-1997) and HUNT3 (2006-2008), participants attending HUNT2 included in a prospective nested case-control design to assess the utility of circulating microRNAs (miRs) to predict future fatal acute myocardial infarction (AMI) in healthy participants as endpoint (I21, ICD-10), 112 cases/control included In the derivation cohort and 100 apparently healthy men (n = 56) and women (n = 44) included in a separate validation cohort, with 10-year follow-up, participants aged 40-70 years.[42]
43	The study was embedded within the Rotterdam Study, a prospective population-based cohort of persons aged 55 years or older in the municipality of Rotterdam, the Netherlands, to assess whether newer risk markers for CHD risk prediction and stratification improve Framingham risk score (FRS) predictions, 5933 participants (men & women), recruitment in (1997-1999) and (2000 -2001), 6.8 years follow-up.[43]
44	10612 Swedish participants (men& women) of five separate sub-studies with availability of DNA and data on cardiovascular risk factors that have been conducted within the registry (SATSA, OCTO-Twin, GENDER, HARMONY and TwinGene), the study aim to compare several multilocus genetic risk score (MGRS) and CHD in 1886 -2000 &1920 -1924, aged 44-≤80 years, 4.3 years follow-up.[44]
45	1153 Participants of the Second Northwick Park Heart Study (NPHS-II), healthy UK men, selected randomly in nested case-control design, aged (50-61 years), who were free of myocardial infarction at recruitment in 1989, followed for a median of 7.8 years for CHD events.[45]
46	2589 Participants (men& women) of the Hisayama study, Japanese population were enrolled in this study, aged 40 years or older, in 1988-2002, 14 years follow-up.[46]
47	9998 participants of Atherosclerosis Risk in Communities (ARIC), cohort study from US, Whites (men& women), subject whom the 9p21 genotype and conventional risk factor information was available included, 13.5 years' follow-up, aged 45-64, recruitment in (1987-1989).[47]
48	A total of 2072 healthy Canadian men were included, free of CHD from the Québec Cardiovascular Study at entry and followed for 13 years, aged 35-64 years from 1990-1991 and in 1998, participants were contacted by mail and invited to participate in this study.[48]
49	23918 healthy Korean men was followed until 2010 in cohort consisted of participants who had visited the Health Promotion Center at Kangbuk Samsung Hospital for a medical check-up in 2005, aged more than 30 years, followed for 5 years.[49]
50	Two UK, Caerphilly and Speedwell population from two neighboring health centers totaling 4860 men were screened for evidence of IHD in between (1979-1983). Men were followed over 10 years and validated coronary events were recorded.[50]
51	Coronary Artery Risk Development in Young Adults (CARDIA) Study, were recruited in 4 U.S. cities, it is a prospective cohort study in which 571 American Black men and 791 women, aged 33-45 years, in (1985-1986), 15-year follow-up.[51]
52	A total of 1135 healthy men and women, aged 33-69 years, participants in the ADVANCE (Atherosclerotic Disease, Vascular FunctioN and GenetiC Epidemiology) study at Kaiser Permanente Northern California and Stanford University were selected, all participants included in this study were free of CVD at baseline and attended comprehensive baseline clinic visits in 2002-2003, 11.3-year follow-up.[52]

53	Three different prospective studies were compared, Multi-Ethnic Study of Atherosclerosis (MESA), Heinz Nixdorf Recall Study (HNR) and the Dallas Heart Study (DHS). In total, 6726 of MESA participants (men& women), aged 45-84, who identified themselves as white, African-American, Hispanic, or Chinese were recruited from six U.S. communities from 2000-2002, they were free of clinical heart disease at baseline and followed for 10 years, the study validated in two independent longitudinal cohort studies included, the Heinz Nixdorf Recall Study, 3692 participants aged 45-75 years, Caucasians, participants from three neighboring cities in Germany, recruited in between the years 2000-2003. The Dallas Heart Study is a multi-ethnic, population-based probability sample of Dallas County, Texas, 1080 participants, aged 45-65, Caucasians, African Americans, and Hispanics, Participants were followed for a median of 9.3 years, the initial data collection was performed in 2000-2002.[53]
54	The CMCS cohort included 30 121 Chinese adults aged 35-64 years at baseline. Participants (men& women) were recruited from 11 provinces and were followed up for new CHD events from 1992-2002. Participants in the Framingham Heart Study were 5251 white US residents of Framingham, Mass, who were 30-74 years old at baseline in 1971-1974 and followed up for 12 years.[54]
55	The Baltimore Longitudinal Study of Aging (BLSA), included 152633 participants (men& women) from two community-dwelling cohort studies in the U.S. and Europe, Caucasian, healthy, well-educated, middle- to upper-middle-class, aged 30-74, mean follow-up of 7.5 years, recruits in 1985.[55]
56	The TARF Study is a longitudinal population-based cohort study (Turkey), 2232 participants (men& women), 30-74 middle-aged adults free of CHD at baseline, were recruited from randomly selected communities in 2002-2003, followed over 7.6 years.[56]
57	Two prospective studies were compared; PMRP and MESA, 1084 participants (men & women), initially CHD-free, selected randomly from Marshfield Clinic Personalized Medicine Research Project (PMRP), a population-based sample repository collected, aged 45-84, recruited in 2002-2004, 5 year follow-up and Multi-Ethnic Study of Atherosclerosis (MESA), 623 participants (men & women), aged 40-80 years old, self-identified as White, African-American, Hispanic, or Chinese, 5.4 year follow-up, All participants were free of cardiovascular disease at study entry (2000-2002).[57]
58	The Tehran Lipid and Glucose Study (TLGS) is a prospective ongoing study aimed at determining the risk factors and the study population consisted of 2568 women aged ≥30 years, free from CHD symptoms at study entry 1999-2001, for a median of 9.3years follow-up.[58]
59	A total of 5271 healthy Korean (men & women), from the third Korea National Health and Nutrition Examination Survey included in this study, aged 20-78, recruitment in in 2005, 13.7-year follow-up.[59]
60	5533 adults (men & women), from the prospective Whitehall II cohort study, UK, were selected using a stratified, multistage probability sampling design to examine whether adding information on job strain to the Framingham model improves its predictive power in a low-risk working population, their aged 35–55, adults were ascertained in Phases 1 (1985-88), 2 (1989-90) and 3 (1991-93), who were CHD free at baseline, 11.3 year follow-up.[60]
61	A total of 29854 men from the Aerobics Center Longitudinal Study, who received a baseline examination from 1979-2002 were included to examine the association of cardiorespiratory fitness (CRF) with risk of coronary heart disease, aged 30-74 years, 12-year follow-up.[61]

62	4903 Participant (men & women), aged 50-70 years from of the St. Francis Heart Study, New York population, USA were included, they had no history, symptoms, or signs of ASCVD between July 1996 and March 1999, 4.3 years follow-up.[62]
63	18225 participants in the Health Professionals Follow-up Study, selected randomly to participate in a nested case-control study among men who were free of diagnosed cardiovascular disease at the time of blood collection, aged 40 -75 years, 6 years of follow-up between1993 and 1995.[63]
64	6606 members (men & women) of the Multi-Ethnic Study of Atherosclerosis (MESA), whites, African American, Hispanic, and Chinese participants., aged 45–84 years, 11.2 years of follow-up, recruitment in 2000- 2002.[64]
65	10 741 individuals (men & women) from 6 European cohorts were included in this stud to evaluate the association between circulating (141) metabolites and incident CHD, randomly selected to participate in case-cohort study, they had no history of myocardial infarction, stroke, heart failure, and atrial fibrillation, 9.2 years of follow-up.[65]
66	A total of 743 participants selected randomly from REGICOR cohort study were included, aged 35–74 years, 6.1 years of follow-up.[66]
67	24443 participants from the MDCS (Malmö Diet and Cancer Study) in southern Sweden included to investigate whether the genetic prediction of CHD differs according to the smoking behavior, 19.4 years follow-up.[67]
68	3559 individuals (men & women) from Taiwan's 2002 Triple High Survey selected randomly 9.7 years follow-up, in 2002.[68]
69	A total of 11242 subjects from GERA cohort study participated randomly to examine the clinical utility of two multi-locus genetic risk scores (GRSs) previously validated in Europeans, aged 30–79 years, 8.7 years of follow-up.[69]
70	3203 individuals selected randomly from TARF cohort study of turkish population, 9.93 years follow-up, recruitment in 2002-2003 and 2007-2008.[70]
71	5398 participants from ARIC cohort study (Atherosclerosis Risk in Communities) were included to detect the CHD by the alterations in the serum metabolome, 30 years of follow-up, aged 45–64 years from 4 US communities, Metabolomic measured in 1997, 2010 and 2014.[71]
72	4679 participants of MESA cohort study were included, aged 45–84 years, 12.5 years of follow-up, recruitment in 2000- 2002.[72]

Table S3: coronary heart diseases definitions as described in the models reviewed.

No	Outcome definition	Frequency
1	International Classification of Diseases, Ninth Revision, codes 410 to 414 or International Classification of Diseases, Tenth Revision, codes I22 to I25.	16
2	International Classification of Diseases 10th Revision (acute myocardial infarction, code I21).	2

3	Hospitalization or death with any of the following primary diagnoses: acute MI and unstable angina (ICD-10: I20.0, I21, I22; ICD-9: 410, 411B; ICD-8: 410, 411 and surgical codes: FNG02, FNG05, FNC, FND, FNE).	2
4	'Hard' CHD events, comprising acute myocardial infarction, sudden death and other coronary deaths, non-fatal CHD events, defined according to the International Classification of Diseases 10th Revision (acute myocardial infarction, code I21).	3
5	International Classification of Diseases, 9th edition (ICD-9) codes 410–414 or ICD-10 codes I20–I25 were present on the death certificate. Non-fatal CHD included first non-fatal MI or first definite angina. Non-fatal MI was defined following MONICA criteria18 based on study electrocardiograms (ECGs), hospital acute ECGs and cardiac enzymes. Incident angina was defined based on clinical records and nitrate medication use, excluding cases based solely on self-reported data without clinical verification and participants with definite angina at baseline.	2
6	International Classification of Diseases (ICD)-9 codes consistent with non-fatal or fatal AMI (410.x), angina pectoris (411.1, 413.x), CHD (414.x), coronary revascularization procedures (CPT4 codes 33510, 33511, 33512, 33513, 33514, 33515, 33516, 33517, 33518, 33519, 33521, 33522, 33523, 33530, 33533, 33534, 33535, 33536, 92980, 92981, 92982, 92984, 92995, 92996) or CHD death (ICD-9 codes 410–414 or ICD-10 codes I20-I25).	1
7	Acute myocardial infarction (MI), old (recognized and unrecognized) MI, angina pectoris, and CHD death. The International Classification of Disease (ICD-8) codes for diseases of the circulatory system	2
8	Acute myocardial infarction (MI), silent MI, sudden cardiac death within 1 hour after the onset of acute illness, or coronary artery disease followed by coronary artery bypass surgery or angioplasty.	1
9	Acute myocardial infarction (MI), silent MI or undergoing coronary surgery.	3
10	Acute MI, coronary death, hospitalization for angina, or coronary revascularization (angioplasty of coronary arteries and coronary artery bypass graft surgery).	1
11	Myocardial infarction or coronary death, CHD death was defined as the absence of non-atherosclerotic cause of death and1 or both of the following: chest pain within 72 hours of death or history of chronic ischemic heart disease in the absence of valvular heart disease or non-ischemic cardiomyopathy.	1
12	Myocardial infarction, fatal CHD, or cardiac procedure.	2
13	Sudden coronary death, fatal acute myocardial infarction, and nonfatal acute myocardial infarction.	1
14	Myocardial infarction as the presence of 2 of the following 3 factors: (1) prolonged chest pain prompting hospital admission, (2) diagnostic evolutionary ECG changes, and (3) elevation of serum creatine kinase to twice the upper limits of normal or a positive serum creatine kinase MB fraction.	1

15	Myocardial infarction , resuscitated cardiac arrest, definite angina (symptoms of typical chest pain and physician diagnosis of angina followed by coronary artery bypass grafting and percutaneous transluminal coronary angioplasty (PTCA), evidence of ischemia by stress tests or resting electrocardiogram, or ≥70% obstruction on coronary angiography), and probable angina.	1
16	Nonfatal myocardial infarction or coronary death (corresponding to ‘‘hard’’ events, as defined in the current FRS, and hospitalization for angina or revascularization (coronary angioplasty or surgery).	1
17	Fatal or non-fatal myocardial infarction, stable or unstable angina, percutaneous coronary revascularization, or bypass grafting.	11
18	Fatal or non-fatal CHD event, which included definite and possible acute MI or coronary death, unstable angina pectoris, revascularization, and unclassifiable fatal events.	5
19	first-ever acute myocardial infarction (MI), silent MI, sudden cardiac death within 1 hour after the onset of acute illness, or coronary artery disease followed by coronary artery bypass surgery or angioplasty.	1
20	Definite or probable hospitalized myocardial infarction or a definite CHD death.	2
21	Angina pectoris, coronary insufficiency, myocardial infarction, and death due to CHD.	1
22	Definite or probable myocardial infarction, silent myocardial infarction (indicated by electrocardiogram), definite CHD death, or coronary revascularization).	4
23	Definite nonfatal or fatal myocardial infarction or death due to CHD. Definite and possible fatal CHD were coded by using the definitions applied within the Cardiovascular Health Study.	1
24	Stable or unstable angina or coronary revascularization procedures (coronary bypass or percutaneous intervention), or death because of CHD, defined according to the International Classification of Diseases-Ninth Revision and International Classification of Diseases-Tenth Revision codes used in event ascertainment.	2
25	Recognized or unrecognized MI, angina pectoris, coronary insufficiency, or CHD death.	2
26	IHD death, clinical non-fatal (definite acute) MI and electrocardiographic MI, as previously described. A major IHD event was defined as one or more of the three possible outcomes described above.	1
27	No reported	2

Table above described the coronary heart diseases outcomes as described by (Damen et. al, 2016)

Table S4: Outcome's categories of CHD used for models reviewed.

No	Outcome category	No	%
1	CHD incident	42	58
2	Fatal and non-fatal myocardial infraction	12	17
3	Acute CHD events, coronary artery revascularization procedures, and silent myocardial infarctions.	2	3
4	Definite or probable MI's, CHD deaths, coronary revascularizations, silent [ECG-confirmed] MI's)/major or minor ECG abnormalities	2	3
5	CHD deaths, non-fatal MI, angina-driven revascularizations, resuscitated cardiac arrests	7	10
6	Death of CHD	1	1
7	CHD events included cases of surgery for angina pectoris	2	3
8	Fatal cardiovascular disease and nonfatal cardiovascular disease	1	1
9	No reported	3	4
Total		72	100

Table S5: Modelling method used to develop the prediction models.

No	Modelling method	Frequency	%
1	Cox proportional hazards regression/ Weibull	47	65
2	Logistic regression/ stepwise	17	24
3	Conditional logistic regression	4	5
4	Lifetime survival analysis	2	3
7	No report	2	3
Total		72	100

Table S6: Method for selection of predictors in models included

No	Predictor's selection	Developed	Validation	Total
1	Imputation	5	1	6
2	Backward approach	3	0	3
3	Forward approach	2	1	3
4	Bayes information criterion (BIC)	6	3	9
5	Akaike information criterion	2	3	5
6	Grambsch and Therneau statistic	2	0	2

7	Schoenfeld test	5	2	7
8	Likelihood ratio test (LR)	9	3	12
9	Shrinkage/penalization	1	2	3
Total		35	15	50

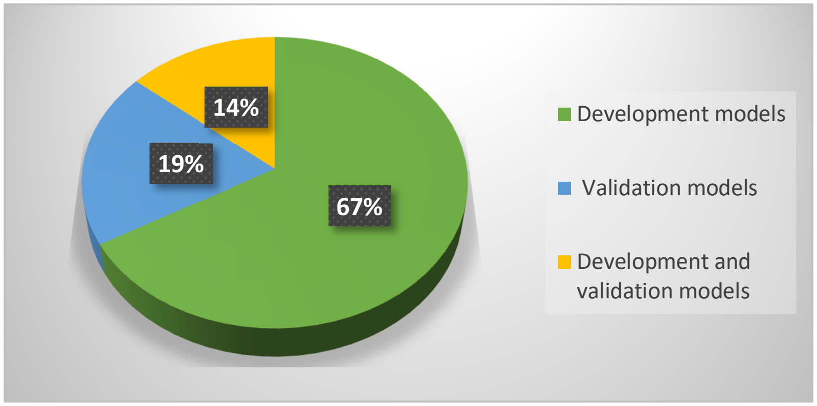

Figure S3: Types of Prediction Modelling Studies identified

The figure above-described types of the models included in the review; most (n=48, 67%) of the model was internal validated studies included (n=10) genetic model, (n=10) 19% is model development and validation (n=5) were genetics, while (n=14) 14% was external validation studies (n=2) were genetics.

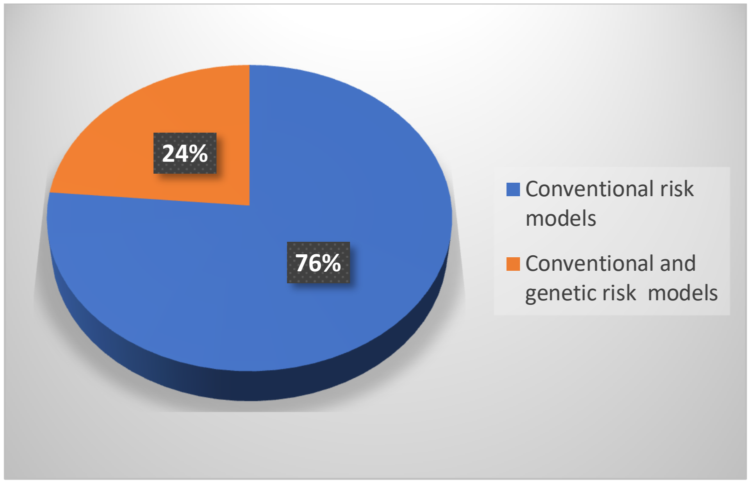

Supplementary Figure S4: The conventional and genetics risk models included

The conventional and genetics risk models included, (n=55, 76%) conventional risk modelling studies while (n=17, 24%) genetic models were included.

Table S7. SNPs included in the genetics modelling studies.

No	SNPs' ID	Gene	Freq	No	SNPs' ID	Gene	Freq
1	rs7412	APOE	1	48	rs2954029	unknown	4
2	rs429358	APOE	1	49	rs1333049	unknown	4
3	rs11591147	PCSK9	1	50	rs3217992	CDKN2B, CDKN2B-AS1	4
4	rs10757274	CDKN2B-AS1	4	51	rs579459	unknown	4
5	rs599839	PSRC1	3	52	rs2505083	JCAD	5
6	rs10455872	LPA	3	53	rs501120	unknown	6
7	rs17465637	MIA3	4	54	rs2047009	unknown	4
8	rs9818870	MRAS	6	55	rs2246833	LIPA	3
9	rs1746048	unknown	4	56	rs974819	unknown	5
10	rs328	LPL	1	57	rs9326246	unknown	2
11	rs7025486	DAB2IP	1	58	rs3184504	SH2B3	7
12	rs1801177	LPL	1	59	rs9319428	FLT1	4

13	rs3798220	LPA	4	60	rs7173743	unknown	4
14	rs662799	APOA5	1	61	rs12936587	unknown	5
15	rs708272	CETP	1	62	rs2281727	SMG6	3
16	rs4341	ACE	1	63	rs15563	UBE2Z	2
17	rs1042031	APOB	1	64	rs2075650	TOMM40	2
18	rs1799983	NOS3	1	65	rs445925	APOC1	3
19	rs17228212	SMAD3	1	66	rs9982601	unknown	5
20	rs7692387	GUCY1A1	5	67	rs10507391	ALOX5AP	1
21	rs17114036	PLPP3	12	68	rs17222842	unknown	1
22	rs12413409	CNNM2	5	69	rs9315050	ALOX5AP	1
23	rs1122608	SMARCA4	7	70	rs17216473	ALOX5AP	1
24	rs9515203	COL4A2	5	71	rs3008621	MIA3	1
25	rs9369640	PHACTR1	2	72	rs646776	CELSR2	3
26	rs11556924	ZC3HC1	5	73	rs3127599	LPAL2	1
27	rs602633	unknown	3	74	rs7767084	LPA	1
28	rs1412444	LIPA	1	75	rs10755578	LPA	2
29	rs4845625	IL6R	4	76	rs2259816	HNF1A	3
30	rs11206510	unknown	5	77	rs6922269	MTHFD1L	1
31	rs17464857	TAF1A, TAF1A-AS	1	78	rs3900940	MYH15	2
32	rs67258870	DHRSX	6	79	rs1010	VAMP8	2
33	rs515135	unknown	3	80	rs7439293	PALLD	2
34	rs2252641	TEX41, LOC100505498	3	81	rs2298566	SNX19	2
35	rs1561198	VAMP5	4	82	rs10797416	SKI	1
36	rs6544713	ABCG8	4	83	rs1490738	PKN2-AS1	1
37	rs1878406	unknown	3	84	rs4268379	SARS1	1
38	rs273909	SLC22A4, MIR3936HG	5	85	rs12127701	MYBPHL	1
39	rs12190287	TCF21	5	86	rs7515901	MYBPHL	1
40	rs2048327	SLC22A3	5	87	rs11806316	unknown	1
41	rs12526453	PHACTR1	5	88	rs11204666	ADAMTSL4-AS1	1
42	rs10947789	KCNK5	4	89	rs12125501	NME7	1
43	rs4252120	PLG	5	90	rs6700559	DDX59-AS1	1
44	rs12205331	ANKS1A	2	91	rs2292096	CAMSAP2	1
45	rs2023938	HDAC9	4	92	rs2820315	LMOD1	1

46	rs12539895	COG5	1	93	rs16986953	unknown	1
47	rs264	LPL	3	94	rs7561273	MFSD2B	1
95	rs10495907	unknown	1	144	rs7074064	BMPR1A	1
96	rs816889	RND3	1	145	rs11203042	LIPA	1
97	rs2351524	NBEAL1	1	146	rs11191447	AS3MT, BORCS7-ASMT	1
98	rs2571445	TNS1	1	147	rs12765878	STN1	1
99	rs4566357	COL4A4	1	148	rs93139	SWAP70	1
100	rs11718455	unknown	1	149	rs7116641	HSD17B12	1
101	rs11710224	LRRC2	1	150	rs12801636	PCNX3	1
102	rs7642590	MAP4	1	151	rs590121	SERPINH1	1
103	rs11916151	unknown	1	152	rs606452	SERPINH1	1
104	rs1393786	PPP2R3A	1	153	rs683800	DCPS	1
105	rs2306374	MRAS	2	154	rs4762911	unknown	1
106	rs4301033	LINC01214, LOC10798641	1	155	rs4149033	SLCO1B1	1
107	rs17655141	unknown	1	156	rs2681472	ATP2B1	1
108	rs17083481	unknown	1	157	rs6490029	CUX2	1
109	rs17087335	NOA1	1	158	rs3809274	unknown	1
110	rs7356185	USP53	1	159	rs17630235	TRAFD1	1
111	rs1429141	unknown	1	160	rs2891403	RPH3A	1
112	rs4469055	unknown	1	161	rs2244608	HNF1A	1
113	rs6841581	EDNRA	1	162	rs11057841	SCARB1	1
114	rs4690974	unknown	1	163	rs9316753	unknown	1
115	rs2736100	TERT	1	164	rs10507753	unknown	1
116	rs10051876	unknown	1	165	rs11617955	COL4A1	1
117	rs246600	ARHGAP26	1	166	rs7139492	COL4A1	1
118	rs2294461	LY86, LY86-AS1	1	167	rs12873154	COL4A1	1
119	rs9472428	PHACTR1, LOC107984015	1	168	rs4773144	COL4A1, COL4A2	3
120	rs883947	PHACTR1	1	169	rs11619057	COL4A2	1
121	rs13211739	PHACTR1	1	170	rs9515201	COL4A2	1
122	rs1321309	unknown	1	171	rs2895811	HHIPL1	4
123	rs3778448	KCNK5	1	172	rs2146238	CYP46A1	1

124	rs4613862	LINC02542	1	173	rs6494488	LOC107984737	1
125	rs17062853	TARID	1	174	rs2487928	JCAD	1
126	rs12663498	PLEKHG1	1	175	rs11072794	LOC105370913,LOC112268142	1
127	rs6926458	LPA	1	176	rs7181240	unknown	1
128	rs1247351	LOC102724087	1	177	rs2880765	AKAP13	1
129	rs972158	SNX10	1	178	rs17514846	FURIN	3
130	rs217	JAZF1	1	179	rs2521501	FES	1
131	rs1167800	HIP1	1	180	rs7496815	unknown	1
132	rs2395858	COG5	1	181	rs4299203	DRC3	1
133	rs4591971	unknown	1	182	rs2071167	UBT, LOC101926967	1
134	rs10237377	PARP12	1	183	rs16948048	ZNF652, LOC102724596	1
135	rs6984210	BMP1	1	184	rs4793721	CA10	1
136	rs17485781	NUGGC	1	185	rs2070783	PECAM1	1
137	rs10962774	unknown	1	186	rs4410190	unknown	1
138	rs16905599	CDKN2B-AS1	1	187	rs892115	SPC24	1
139	rs10965228	CDKN2B-AS1	1	188	rs17318596	DMAC2	1
140	rs495828	unknown	1	189	rs2288911	APOC2, APOC4-APOC2	1
141	rs11238956	unknown	1	190	rs8111989	CKM	1
142	rs17155842	unknown	1	191	rs6088638	ACSS2	1
143	rs3748242	ANXA11	1	192	rs867186	PROCR, MMP24-AS1-EDEM2	1
193	rs2832227	MAP3K7CL	1	212	rs4994	ADRB3	1
194	rs1034565	ARVCF	1	213	rs12102203	DMXL2	1
195	rs9608859	unknown	1	214	rs1122955	ZNF132	1
196	rs17609940	ANKS1A	2	215	rs1799963	F2	1
197	rs10953541	BCAP29, DUS4L-BCAP29	2	216	rs2961135	OR2A25, LOC105375548	1
198	rs964184	ZPR1	1	217	rs89962	KRT5	1
199	rs10757274	CDKN2B-AS1	1	218	rs10822891	CTNNA3	1
200	rs2383206	CDKN2B-AS1	1	219	rs4796603	HAP1	1
201	rs17011666	MIA3	1	220	rs1800437	GIPR	1
202	rs3825807	ADAMTS7	2	221	rs3749817	FSTL4	1
203	rs4380028	unknown	1	222	rs8089	THBS2, LOC101929523	1
204	rs2228671	LDLR	1	223	rs4977574	CDKN2B-AS1	1

205	rs7278204	unknown	1	224	rs216172	SMG6	1
206	rs20455	KIF6, LOC107986594	2	225	rs46522	UBE2Z	1
207	rs11016076	MKI67	1	226	rs318090	UBE2Z	1
208	rs2213948	unknown	1	227	rs2028900	MAT2A, PARTICL	1
209	rs2296436	HPS1	1	228	rs4299376	ABCG8, LOC102725159	1
210	rs428785	ADAMTS1	1	229	rs11984041	HDAC9	1
211	rs402007	ADAMTS1	1	212	rs4994	ADRB3	1

Table S8. PICOTS elements of the articles reviewed.

No	Authors	Countries	Population (Free from CHD)	Sample	Intervention and Comparator	Outcome (Incident of CHD)	Times Follow-Up	Study Design
1	Iribarren et al., 2016	Spain	GERA, white non-Hispanic	51,954	GRS + CRFs and CRFs	1864; 1077 male/ 787 female	5.9	Cohort
2	Hughes et al., 2012	UK	MORGAM, Caucasian	4818	GRS + CRFs and CRFs	1736; 632 cases, 1361 non-cases	18	Case-cohort
3	Talmud et al., 2008	UK	NPHSII, Caucasian	2742 Male	GRS + CRFs and CRFs	270 male	15	Cohort
4	Humphries et al., 2006	UK	NPHSII, Caucasian	2057 Male	GRS + CRFs and CRFs	183 male	10.8	Cohort
5	Beaney et al., 2017	UK	NPHSII, Caucasian	2075 Male	GRS + CRFs and CRFs	284 male	13.5	Cohort
6	Antiochos et al., 2016	Switzerland	Colaus, Caucasian	4283	GRS + CRFs and CRFs		5.6	Cohort
7	Brautbar et al., 2012	USA	Non-Hispanic white	1.8542<br>2.2068<br>3.2339	GRS + CRFs and CRFs in 1. ARIC, 2. Rotterdam, 3. FRS	1.1110<br>2.2068<br>3.2339	1.18<br>2.10<br>3.10	Cohort
8	Chien et al., 2007	China	Chin-Shan	3568	Derived, CRF-based	122 (79 male/ 43 female)	13.6	Cohort

9	Simmons et al., 2008	UK	Norfolk	10,295	Derived, CRF-based	430 male, 250 female	8.5	Cohort
10	Macleod et al., 2006	UK	Scottish men	5191	Derived, CRF-based	203 deaths, 200 hospitalized	15	Cohort
11	Nishimura et al., 2014	Japan	Japanese urban (Suita)	5521	Derived, CRF-based	213	11.8	Cohort
12	Ingelsson et al., 2007	Boston	Framingham, Massachusetts	3322	Derived, CRF-based	291, 198 male	15	Cohort
13	Cao et al., 2017	USA	MESA	4679	Derived, CRF-based	150 MI, 24 resuscitated cardiac arrest, 70 deaths, and 132 definite anginas	12.5	Cohort
14	Nambi et al., 2009	Houston	ARIC	13145	Derived, CRF-based	1812	15.1	Cohort
15	Cushman et al., 2005	Boston	CHD	3971	Derived, CRF-based	547 MI or deaths	10	Cohort
16	Cooper et al., 2005	UK	NPHSII, Caucasian	3052 Male	Derived, CRF-based on PROCAM	110 male (PROCAM), and 109 male (Framingham)	10.8	Cohort
17	Orford et al., 2002	Boston	Normative aging study	1393	Derived, CRF-based	206 CHD	10	Cohort
18	Jee et al., 2014	Korea	Korean Heart Study	268,315	Derived, CRFs (Framingham)	2596 (1903 nonfatal, 693 fatal)	11.6	Cohort
19	Merry et al., 2011	Netherlands	Dutch CAREMA	21,148	Derived, CRFs (SCORE)	783	10.9	Cohort
20	Khalili et al., 2011	Iran	Urban	5101	Derived, CRFs	387 (169 Female)	9.3	Cohort
21	Auer et al., 2012	Switzerland	Health ABC study	2192	Derived, CRFs Framingham	351 (96 CHD, 101 MIs, 154 anginas)	8	Cohort

22	Taylo et al., 2001	USA	US Army	630	Derived, CRFs Framingham	No information	5	Cohort
23	Rana et al., 2009	UK	EPIC Norfolk	25,663	Derived, CRFs	No information	6	Nested case-control
24	Parikh et al., 2016	USA	Women's health initiative	27,982	Derived, CRFs	4607	12	Cohort
25	De Vries et al., 2015	Netherlands	Rotterdam, Ommoorod	5899	GRS + CRFs and CRFs	904 (460 MIs)	10	Cohort
26	Paynter et al., 2011	Boston	ARIC	12,834	Derived, CRFs	No information	3	Cohort
27	Morrison et al.,2007	Houston	ARIC	15,792	GRS + CRFs and CRFs	1452	13	Cohort
28	Folsom et al., 2007	USA	ARIC	15,792	Derived, CRFs	No information	5	Nested case-control
29	Detrano et al., 1999	USA	South Bay Heart	1196	Derived, CRFs	17 deaths and 29 nonfatal MIs, 4 fatal	3.5	Cohort
30	Tada et al., 2016	Sweden	Malmo diet and cancer	23,595	GRS + CRFs and CRFs	2213	14.4	Cohort
31	Aekplakorn et al., 2007	Thailand	Electricity employees	2536	Derived, CRFs	66	17	Cohort
32	Bolton et al., 2013	Scotland	Edinburgh artery study	840	GRS + CRFs and CRFs	319	15	Cohort
33	Lloyd et al., 2004	USA	Framingham	6216	Derived, CRFs	93 CHD and 1363 died free of CHD.	10	Cohort
34	Empana et al., 2003	France	PRIME, Men (Belfast, and France)	1.2399 2.7359	CRFs (Framingham, and PROCAM)		5	Cohort
35	Rodondi et al., 2012	USA	Health ABC study	2193	CRFs (Framingham older and recalibrated)	351	8	Cohort

36	McGeechan et al., 2008	USA	ARIC	9155	Derived, CRFs	700	8.8	Cohort
37	Onat et al., 1997	Turkey	TARF study	2259	Derived, CRFs	55 deaths, 69 nonfatal coronaries	5	Cohort
38	Wilson et al., 1998	USA	Framingham	5345	Derived, CRFs	383 men and 227 women	12	Cohort
39	Mainous et al., 2008	USA	ARIC	9307	Derived, CRFs	299 men and 131 women	6	Cohort
40	Pyorala et al., 1998	Finland	Helsinki policemen	970	Derived, CRFs	164 men, major CHD event	22	Cohort
41	Hiram et al., 1994	USA	Angiographically controlled study	848	GRS + CRFs and CRFs	No information	3.2	Cohort
42	Bye et al., 2016	Norway	HUNT studies	212	miRS + CRFs and CRFs	No information	10	Nested case-control
43	Kavousi et al., 2012	Netherlands	Rotterdam	5933	Derived, CRFs	347: (190 nonfatal MIs, and 157 CHD deaths)	6.8	Cohort
44	Ganna et al., 2013	Sweden	Swedish	10,612	GRS + CRFs and CRFs	781	4.3	Cohort
45	Cooper et al., 2000	UK	NPHS-II	928	Derived, CRFs	104: (71 acute CHD events, 15 anginas, 18 new major Q waves)	7.8	Nested case-control
46	Arima et al., 2007	Japan	Japanese	2589	Derived, CRFs	129	14	Cohort
47	Brautbar et al., 2009	USA	ARIC	9998	9p21 + CRFs and CRFs	1349	13.5	Cohort
48	St-Pierre et al., 2006	Canada	Quebec cardiovascular study men	2072	Derived, CRFs	230 deaths or nonfatal MIs	13	Cohort

49	Ryoo et al., 2016	Korea	Korean men	23,918	Derived, CRFs	5763	5	Cohort
50	Yarnell et al., 2004	UK	Caerphilly collaborative study	4860	Derived, CRFs	525	10	Cohort
51	Everage et al., 2009	USA	CARDIA	1362	Derived, CRFs	No information.	15	Cohort
52	Iribarren et al., 2015	USA	ADVANCE	1135	Derived, CRFs	164	11.3	Cohort
53	McClelland et al., 2015	USA	1. MESA<br>2. Heinz<br>3. Dallas	1.6726<br>2.3692<br>3.1080	Derived, CRFs	1.422<br>2.274<br>3.58	1.10.2<br>2.10.4<br>3.9.3	Cohort
54	Liu et al., 2004	China	1. CMCS<br>2. Framingham	1.30121<br>2.5251	Derived, CRFs	1.192, and 273 Hard 2.625, and 293 deaths.	12	Cohort
55	Brant et al., 2010	USA	1. USA (BLSA)<br>2. Europe (VHD and PP)	1.1966<br>2.150667	Derived, CRFs	2457	7.5	Cohort
56	Onat et al., 2010	Turkey	Turkish	2232	Derived, CRFs	302	7.6	Case-cohort
57	Cross et al., 2012	USA	1. CHDRA<br>2. MESA	1.1084<br>2.6814	Derived, CRFs	179	5, and 5	Case-cohort
58	Hadaegh et al., 2012	Iran	Tehran lipid and glucose study	2568	Derived, CRFs	127	9.3	Cohort
59	Kang et al., 2012	Korea	3rd Korea National Survey	5271	Derived, CRFs	100	13.7	Cohort
60	Kivimaki et al., 2011	UK	Whitehall	5533	Derived, CRFs	160 deaths and nonfatal MIs	11.3	Cohort
61	Gander et. al., 2015	USA	Aerobic centre	29,854	Derived, CRFs	499	12	Cohort
62	Arad et al., 2004	USA	Francis heart study	4613	Derived, CRFs	119	4.3	Cohort

63	Pischon et al., 2005	USA	Health professionals study	18,225	Derived, CRFs	266	6	Nested case-control
64	Polak et al., 2015	USA	MESA	6606	Derived, CRFs	484: (209) angina	11.2	Cohort
65	Cavus et al., 2019	Germany	BiomarCaRE	10,741	Derived, CRFs	2166	9.2	Case-cohort
66	Subirana et al., 2018	Spain	REGICOR	638	Derived, CRFs	105	6.1	Case-cohort
67	Hindy et al., 2018	Sweden	Malmo iet and Cancer Study	24,443	GRS + CRFs and CRFs	3217	19.4	Cohort
68	Chien et al., 2018	Taiwan	Taiwanese	3559	Derived, CRFs	63	9.7	Cohort
69	Iribarren et al., 2018	USA	GERA	11,242	Derived, CRFs	450	8.7	Meta-analysis.
70	Gunayca et al., 2019	Turkey	TARF	3203	Derived, CRFs	573	9.93	Cohort
71	Wang et al., 2019	USA	ARIC	5398	Derived, CRFs	633	30	Cohort
72	Cao et al., 2018	USA	MESA	4623	Derived, CRFs	315	12	Cohort

MI: Myocardial infarctions, GRS: genetic risk score, CRFs: conventional risk factors, GRS: genetic risk score, CRFs: conventional risk factors, NPHS-II: the second Northwick Park heart study.

Table S9. Discrimination, calibration, and risk classification as described in the reviewed models.

No *	Authors	Predictors (Genetics and Biomarkers)	Improvement of the Model's Performance		
			**Discrimination**	**Calibration (Goodness-of-Fit Test)**	**Risk Classification**
1	Iribarren et al., 2016	FRS + 4 constructed GRSs; (GRS_8, GRS_12,	ΔC-statistic: 1. GRS_8b = (0.008), 2. GRS_36 = (0.008), 3. GRS_12 = (0.007), 4. GRS_51 = (0.009), *p* = <0.001 for all models	Hosmer–Lemeshow chi-square >0.20 in all GRS models	NRI: (5%) GRS_8 and GRS_12 and GRS_36, and (4%) for GRS_51.

		GRS_36, and GRS_51) plus			
2	Hughes et al., 2012	2 GRS constructed; (GRS1) combined 11 SNPs + 2 haplotypes + FRS, GRS2 combined 11 SNPs plus 4 SNPs + FRS.	Yes, marginally, only in GRS2, C index improvement 1.11%, $p = 0.048$, no significant discrimination improvement for GRS1 0.752, $p = 0.11$.	-	Yes (both score) NRI = 7.5%, $p = 0.017$ for GRS1, 6.5%, $p = 0.044$ for GRS2
3	Talmud et al., 2008	9p21.3 (rs10757274 + 10 models SNPs) + CRFs	Yes, partially, AUC (rs10757274 + CRFs) = 0.64, $p = 0.14$. AUC for (CRFs + 10 models SNPs); addition of 1 SNP, $p < 0.03$, addition of 2 or more; $p = < 0.001$.	Hosmer–Lemeshow	Yes (event group to moderate risk 13.5% and 3.3%)
4	Humphries et al., 2006	12 genes + CRFs	Yes, AUC = 0.62, (0.58–0.66) [12.6% detection rate for a 5% false-positive rate (DR5)] ($p = 0.001$).	likelihood ratio	-
5	Beaney et al., 2017	GRS constructed; 19 SNP + QRISK2 compared to QRISK2 alone and 21 SNPs + QRISK2	Yes, partially, 19 SNPs (AUC) = (0.68 vs. 0.70 $p = 0.02$), and 21 SNPs with no significant improvement in discrimination ($p– = 0.55$)	1. 19 SNPs had good calibration ($p = 0.17$), and 21 SNPs were poorly calibrated ($p– = 0.03$)	Yes; 19 SNPs, the NRI = (0.07, $p = 0.04$), QRISK alone = (0.17), 21 SNPs no improvement in net reclassification ($p = 0.10$)
6	Antiochos et al., 2016	Parental history (PH) + GRS (153SNPs) + CRFs	Yes, (GRS + CRFs); C index improvement = 0.016, $p = 0.048$. GRS + CRFs + PH; C index improvement = 0.022, $p = 0.006$.	Hosmer–Lemeshow, $0.35 < p < 0.94$	Yes, NRI was significant, IDI was significant
7	Brautbar et al., 2012	GRS (13SNPs) + CRFs in 3 groups	Yes, (CRFs + GRS); AUC Unweighted GRS:1. ARIC: (0.742–0.749), 2. Rotterdam: (0.729–0.734), 3. Framingham:	Grønnesby–Borgan for derived and based 1. ARIC:	NRI: Unweighted GRS: 1. ARIC: 6.3%, 2. Rotterdam: 0.2%,

			(0.773–0.775). Weighted GRS: 1. ARIC: (0.742 to 0.751), 2. Rotterdam: (0.729–0.735), 3. Framingham: (0.773–0.784)	(p-value (= 0.05), and (0.003). 2. Rotterdam: $p > 0.4$) for both. Rottedam: $p > 0.4$) for both.	3. Framingham: −0.6% Weighted GRS: 1. ARIC: 7.3%, 2. Rotterdam: 3.6%, 3. Framingham: 4.5%
8	Chien et al., 2007	CRFs + (ApoB,non-HDLC, LDLC)	Yes, AUC (ApoB = 0.63)	Hosmer–Lemeshow	-
9	Simmons et al., 2008	CRFs + glycated hemoglobin	Yes, AUC for CRF-based = 0.72 for males, 0.80 for females. AUC new model = 0.73 and 0.80.	-	NRI was 3.4% male and 2.2 % female
10	Macleod et al., 2006	CRFs + psychosocial risk factors	Yes, AUC: 1 = 0.754, 2 = 0.745, 3 = 0.746, 4 = 0.746, 5 = 0.749	-	Recalibration coefficient based upon 20 iterations
11	Nishimura et al., 2014	CRFs + TC, LDLC + Suita with/without CKD	ROC for LDL Suita + CKD = (0.831), TC Suita + CKD = (0.835), Suita with/without CKD = (0.833 and 0.835)	Bayesian information criteria (BIC) and likelihood ratio.	NRI and IDI: markedly for TC (Suita) + CKD, NRI for TC Suita + CKD (46.8%, $p < 0.001$)
12	Ingelsson et al., 2007	CRFs + lipids (TC, LDLC, HDLC, ApoA, ApoB	No, C index of ApoA, ApoB model in both men and women (0.74, 0.76), $p > 0.70$	Likelihood ratio	Was not statistically significant
13	Cao et al., 2017	CRFs + ApoB	-	-	-
14	Nambi et al., 2009	CRFs + C-IMT + Plaque	Yes, AUC; CRFs = (0.674), others (0.690, and 0.686) in men, AUC = (0.759 to 0.762 and 0.770) women.	Grønnesby–Borgan, CRFs. Partially good fits in women	NRI, clinical NRI, CRFs + CIMT + Plaque model was better
15	Cushman et al., 2005	CRFs + CRP + CIMT	-	-	-

16	Cooper et al., 2005	CRFs + LDL, HDL (PROCAM, and FRS)	ROC = 0.6295 (PROCAM) and 0.6184 (FRS)	Hosmer–Lemeshow, 0.46 (PROCAM), and 0.47 (FRS) $p <$ 0.0001 for both	-
17	Orford et al., 2002	CRFs (Framingham, and European)	C-statistic: 1. FRS (0.60), 2. EUR(0.58),	-	-
18	Jee et al., 2014	CRFs + LDL, HDL, TG (3 models)	AUC:<br>1. Male: (0.764, 0.758, and 0.757)<br>2. Female: (0.812, 0.809, and 0.815).	Hosmer–Lemeshow	NRI = (0.284, 0.185, and 0.109) in male, and (0.177, 0.160, and 0.207) in female.
19	Merry et al., 2011	CRFs (re-estimated SCORE) + Other factors (total, HDL) in 9 models	AUC: CAREMA (0.802), re-estimated SCORE high and low (0.789).	Calibration slope and intercept: CAREMA (1.00, 0.07%), re-estimated SCORE high and low (2.32, 3.29%), and (4.63, 4.19%).	NRI: CAREMA (28%), re-estimated SCORE high and low (28%), and (35%) respectively.
20	Khalili et al., 2011	CRFs + Psychosocial risk factors (Rose + ECG)	C-statistic:<br>1. Male (0.713) for Rose, and (0.717) for Rose + ECG, p = 0.179.<br>2. Female (0.770) for Rose, and (0.786) for Rose + ECG, p = 0.09.	-	Integrated discrimination improvement:<br>1. Male (4.01%) p (0.537).<br>2. Female (8.78%) p (0.309).
21	Auer et al., 2012	CRFs + ECG	C-index: 0.58	Hosmer–Lemeshow, calibration: p = 0.01, and p = 0.03	NRI: derived and based: 7.4% and 57%, IDI: 0.99% and 1.03%
22	Taylo et al., 2001	CRFs + CAC using EBCT	ROC = 0.62, $p < 0.001$, and 0.61, $p < 0.001$.	-	-

23	Rana et al., 2009	CRFs + CRP + myeloperoxidase in (8) models	C-statistic for derived and based = (0.65_0.54).	Hosmer–Lemeshow did not perform well.	NRI: derived and based on 27.4%, and 6.4%
24	Parikh et al., 2016	CRFs + reproductive factors	C-statistic for derived and based 1. Based (0.726) and derived (0.730)	-	IDI: 0.0013 ($p <$ 0.0001), and 0.002 ($p =$ 0.04).
25	De Vries et al., 2015	GRS (152 SNPs) + CRFs in (3) models	C-statistic: 1.0.684, 2. 0.716, 3.0.716	-	NRI: derived and based:1. 0.034–0.003, 2.0.01–0.022, 3.0.007–0.017
26	Paynter et al., 2011	CRFs + (SBP, HDL, and TC) in (3) models	AUC: 1. Male: 0.701, 0.704, and 0.704, 2. Female: 0.780, 0.785, and 0.785	Hosmer–Lemeshow, 1. Male: 12.4 (0.14), 12.3 (0.14), and 13.5 (0.10), 2. Female: 2.1 (0.98), 5.7 (0.68), 13.6 (0.09).	NRI: derived and based, 1. −1.1% (0.47), −0.2% (0.55), 2. 6.4% (0.008), 5.4 (0.016)
27	Morrison et al.,2007	GRS (116 SNPs) + CRFs.	AUC: 1. White (GRS + CRFs) = 0.766, and 0.764 for CRFs alone. 2. Black: (GRS + CRFs) = 0.769, and 0.758 for CRFs alone.	-	-
28	Folsom et al., 2007	CRFs + 19 novel risk markers included CRP	AUC: 1. Basic: (0.767–0.820) 2. Derived: (0.768–0.824)	-	-
29	Detrano et al., 1999	CRFs + EBCT Calcium score in (4) models	ROC: derived and based 1. Basic: 0.64 ± 0.05 2. FRS: 0.69 ± 0.05 3. Data derived: 0.68 ± 0.05 4. Derived + Ca: 0.71 ± 0.04	-	-
30	Tada et al., 2016	GRS (27, and 50 SNPs) + CRFs + FH in (4) models	C-statistic for derived and based (+GRS27, +GRS50, and +FH): 1. Established risk factors: (0.746, 0.748, 0.749, and 0.749)	-	NRI: derived and based: 1. Established risk factors: 0.20% ($p <$ 0.001); 2. Established

			respectively; 2. Established risk factors + FH: (0.749, 0.751, 0.752, and NA), respectively.		risk factors + FH + 27GRS = 0.15% ($p <$ 0.001), 3. Established risk factors + FH + 50GRS = 0.17%, $p <$ 0.0001
31	Aekplakorn et al., 2007	CRFs + BMI + WC, and waist-to-hip ratio, wais-to-height ratio in (4) models	AUC: 1.0.606, 2.0.627, 3.0.592, 4.0.651	-	-
32	Bolton et al., 2013	GRS (27 SNPs) + CRFs, (4) models for SNPs identified in GWAS and (2) models for SNPs identified in regression trees.	C-index: SNPs identified from (GWAS, and regression trees): (a) GWAS: 1. CHD: 0.761, 0.740, 0.671 and 0.741; 2. Fatal or nonfatal MI or coronary intervention: 0.717, 0.750, 0.718, and 0.753. (b) Regression trees: 1. CHD: 0.686 and 0.709 2. Fatal or nonfatal MI or coronary intervention: 0.694 and 0.718.	-	NRI plus IDI: (a) GWAS: 1. CHD: NRI = 54.4 for both models, IDI = 0.04 for both models, 2. Fatal or nonfatal MI or coronary intervention: NRI = 43.5 and 42.7, IDI = 0.05 for both models, (b) Regression trees: 1. CHD: NRI = 41.5, IDI = 0.04, 2. Fatal or nonfatal MI or coronary intervention: NRI = 42.9, IDI = 0.03
33	Lloyd et al., 2004	CRFs (Framingham) stratified into age and gender tertiles	Specific index ages: (40–80) per tertiles: 1. Men %: (31.2 -16.1), (33.6–14.4), and (46.8–34.2); 2. Women %: (9.7 -11.), (15.1–19.6), and (25.9–24.9).	-	-

34	Empana et al., 2003	CRFs (Framingham and PROCAM) in Belfast and France	C-statistic in prime populations: 1. FRS: 0.66 in Belfast, and 0.68 in France. 2. PROCAM: 0.61 in Belfast, and 0.64 in France	1. Framingham: common ratios were 1.34 in Belfast and 2.35 in France; 2. PROCAM: 1.78 in Belfast, and 2.76 in France	-
35	Rodondi et al., 2012	CRFs (Framingham) directly and after recalibration	C-index: Framingham older: c-index = (0.583–0.606) in men and (0.577–0.598) in women	Hosmer–Lemeshow, and rift function (FRS, recalibrated FRS, and refit FRS) 1. Men: 16.27–16.11, and 4.89.; 2. Women:121.43, 22.73, and 7.96.	-
36	McGeechan et al., 2008	CRFs + retinal vascular	AUC: 1. Based: 0.695, 2. Derived: 0706	-	-
37	Onat et al., 1997	CRFs + (DBP)	-	-	-
38	Wilson et al., 1998	CRFs + TC (NCEP) + BP (JNC_V)	AUC: 1. Men: ? ?, 2. Women: ?	-	-
39	Mainous et al., 2008	CRFs + (Framingham) at baseline visit and 3 and 6 years before	AUC: (baseline visit and 3 and 6 years before) 1. Men: 0.646, 0.667, and 0.649. 2. Women: 0.667, 0.677, and 0.709. 3. Total: 0.720, 0.727, and 0.730.	-	-
40	Pyorala et al., 1998	CRFs + OGTT + insulin measurements	AUC: (5, 10, 15, and 20) quantiles: 1. Age-adjusted: 3.29, 2.72, 2.14, and 1.61, 2. With	-	-

			AUC glucose: 2.36, 2.29, 1.76, and 1.32.		
41	Hiram et al., 1994	CRFs + 12 SNPs (apolipoprotein)	Stringent criteria to classify patient (>60% stenosis), (<10% stenosis)	-	-
42	Bye et al., 2016	12 miRS + CRFs and CRFs	AUC: derived and baseline 1. 1. 1. Derived: 0.91, 2. FRS 0.72.	-	-
43	Kavousi et al., 2012	CRFs + CAC in (12) models	C-statistic: increased 0.05 by adding CAC score.	Roche Diagnostics, Mannheim, Germany.	NRI: 19.3% CAC score
44	Ganna et al., 2013	GRS (395 SNPs) + CRFs and CRFs in (4) models	C index: 1. Basic model: 0.702. 2. GRS (395 SNPs): Increment = 0.002, 3. CHD (46): Increment = 0.004., 4. polygene: Increment = 0.001	GrønnesbyBorgan: 0.96, 0.99, and 0.73	IDI: 0.2, 0.4, and 0.1
45	Cooper et al., 2000	CRFs + Hemostatic factors	AUC:1. Model a (0.54–0.77), 2. Model b (0.66–0.77)	-	-
46	Arima et al., 2007	CRFs + high sensitivity CRP	Typical symptoms, abnormal cardiac enzymes	-	-
47	Brautbar et al., 2009	9p21 + CRFs	AUC: increased from 0.782 to 0.786.	Grønnesby–Borgan: 20.114, p = 0.0172	NRI: 1. CRFs + 9p21: 0.8% p = 0.3, clinical NRI = 6.2%, p value = 0.03. IDI: 0.002, $p <$ 0.015, 2. CRFs: the clinical NRI 6.8%, IDI: 0.021.
48	St-Pierre et al., 2006	CRFs + ApoB (tertiles), LDLC in (3) models	AUC: Model 1 = 68.9%, Model 2:70.3%, $p < 0.001$ (Base + ApoB), Model 3: 70.7%, $p <$ 0.001 (Base + LDLC)	-	-
49	Ryoo et al., 2016	CRFs + ApoB, ApoA, A/B ratio (Quintile)	-	-	-

50	Yarnell et al., 2004	CRFs + plasma lipid + 3 haemostatic /inflammatory.	AUC: (lipid, haemostatic, and combined): 1. Whole cohort: 0.724, 0.728, and 0.737, 2. Men without CHD: 0.685, 0.693, and 0.707.	Hosmer–Lemeshow, 1. 6.37, $p = 0.61$, 10.46, $p = 0.23$.	-
51	Everage et al., 2009	CRFs + CAC score + Racial	Perceived racial discrimination score (respond to year 7, and 15 plus cumulative score): OR: 0.88, 0.82, and 0.90.	-	-
52	Iribarren et al., 2015	CRFs + cardiac troponin I	AUC: 1. With hs Troponin = 0.7008, 2. without hs Troponin = 0.6849.	-	NRI: 18% after adding hs Troponin to the models
53	McClelland et al., 2015	CRFs + CAC	C-statistic derived and basic models: 1. FRS: 0.750 (MESA), 0.720 (HNR), and 0.782 (DHS). 2. FRS + CAC: 0.800 (MESA), 0.779 (HNR), and 0.816 (DHS).	Hosmer–Lemeshow, $p > 0.22$ for each	Optimal model for CHD risk prediction: Discrimination slope: 1. FRS: 0.052 (MESA), 0.053 (HNR), and 0.046 (DHS).,2. FRS + CAC: 0.086 (MESA), 0.095 (HNR), and 0.078 (DHS).
54	Liu et al., 2004	CRFs (based and after recalibration.	C-statistic: 1. FRS: 0.705 (men), 0.742 (women). 2. CMCS: 0.736 (men), 0.759 (women).	Hosmer–Lemeshow, $p < 0.01$	No information -
55	Brant et al., 2010	CRFs + BP measurements per age and gender.	AUC:<br>1. PP models: 0.83–0.85,<br>2. BP models: 0.77–0.81	Hosmer–Lemeshow, 1. BLSA $p = 0.75$, male, $p = 0.02$ in VHM and PP in (female $p = 0.01$).	
56	Onat et al., 2010	CRFs + MetS + CRP per quintiles	AUC: 1. Derived models: 0.789 in men, and 0.806 in women., $p < 0.001$ for each.	No information -	Men and women in the highest quintiles were significantly and 20–27-fold more

			2. Without CRP: 0.781 in men, and 0.803 in women. $p < 0.001$ for each, 3. FRS: 0.775 in men, and 0.783 in women. $p < 0.001$ for each.		likely to develop CHD than those in the lowest quintiles.
57	Cross et al., 2012	CRFs + 19 biomarkers	AUC: 2. CHDRA = 0.72, Framingham = 0.73, p value = 0.70.	No information -	NRI:1. CHDRA: 25.7% with events, and 17% without events. A clinical NRI = 42.7%, $p < 0.001$. A clinical NRI = 42.7%, p value < 0.001.
58	Hadaegh et al., 2012	CRFs (Framingham) + Electrocardiogram	C-statistic derived and basic models: 1. reestimated FRS = 0.838, 2. FRS + electrocardiogram = 0.844, 3. combined = 0.843.	Hosmer–Lemeshow,	NRI: 1. Based = 6.0%, cut point -free NRI = 20.8%
59	Kang et al., 2012	CRFs (Framingham) + MetS	AUC: Men: FRS (0.767), and (0.677) for FRS + MetS, $p < 0.01$, Women: for FRS (0.777, and (0.733) for FRS + MetS, it was not significant.	-	-
60	Kivimaki et al., 2011	CRFs + job strain	C-statistic derived and basic models: 1. FRS = 0.7252. FRS + job strain = 0.726.	Hosmer–Lemeshow was not significant.	NRI: derived model = 0.7% and 1.0%
61	Gander et. al., 2015	CRFs + cardiorespiratory fitness (CRF)	AUC: derived and FRS = 0.80 for both $p = 0.97$	-	-
62	Arad et al., 2004	CRFs + CRP + CAC score	C-statistic: 1. Without CRP = 0.83, 2. Without CAC score = 0.80, 3. With CAC + CRP = 0085.	-	-

63	Pischon et al., 2005	CRFs + ApoB + non-HDL, LDL	-	Hosmer–Lemeshow	-
64	Polak et al., 2015	CRFs + CCA IMT	C-statistic: 1. Far-wall IMT + CRFs: Increment = 0.012, $p <$ 0.001, mean IMT($p = 0.004$).	$R^2 = 0.31$, 0.26, and 0.22.	NRI: 6%, and 20%.
65	Cavus et al., 2019	CRFs + 141 metabolites	C-statistic: 1. 0.756, 2.0.755, 3 + 4. 0.754.	-	-
66	Subirana et al., 2018	CRFs + 9 biomarkers	C-index: 1. Without biomarker (%) = 1.74.3, 2. 81.3, and 3. 81.3%, 2. With biomarker (%) = 1.79.3, 2. 82.5, 3. 82.6	-	NRI: 33.7%
67	Hindy et al., 2018	CRFs + GRS (50 SNPs), and smoking	AUC: (never, former, and current smokers), 1. FRS:0.747, 0.742, and 0.740.; 2. Derived: 0.797, 0.749, 0.744.	-	IDI: 0.012, 0.006, and 0.004, $p < 0.0001$ for all.
68	Chien et al., 2018	CRFs + (FRS in Taiwanese in (8) models	AUC: ranged from 0.804–0.850 by estimates point, and 0.691–0.847 by predicted risks.	Hosmer–Lemeshow, p (0.10–0.79)	-
69	Iribarren et al., 2018	CRFs + CRFs + GRS (12 and 51 SNPs) in 3 population (AA, LAT, AS).	C-statistic: (GRS_12, GRS_51) 1. AA: 0.687, and 0.687(FRS), and 0.691, and 0.690 (FRS + GRS)., 2. LAT: 0.714, and 0.714(FRS), and 0.717, and 0.715 (FRS + GRS)., 3. AS: 0.745, and 0.745(FRS), and 0.747, and 0.750 (FRS + GRS).	Hosmer–Lemeshow, p values were not significant over all models.	NRI: overall; 10%, 7%, and 7%.
70	Gunayca et al., 2019	CRFs + CRFs + BMI, WC, in (6) models	AUC: (unadjusted models) 1. Men: 0.572–0.600, 2. Female: 0.587–0.652, $p < 0.001$ for both.	-	-
71	Wang et al., 2019	CRFs +	AUC: increased (0.724 to 0.740).	Cross-validation	NRI:0.522, $p < 0.001$, IDI: 0.038, $p = 0.002$

		CRFs + (19) Metabolites			
72	Cao et al., 2018	CRFs + ApoB (Roche, Kamiya, and Diazyme).	-	-	-

* The number refers to supplementary table reference list (see page 18–23 of the Supplementary File). CRFs: Conventional risk factors, FRS: Framingham, EUR: European, AUC: area under receiver characteristic, MIs: myocardial infarction, GRS: genetic risk score, CRFs: conventional risk factors, NRI: net reclassification improvement, IDI: integrated discrimination improvement, EBCT: electron-beam computed tomography, CRP: C-reactive protein, CAC: coronary artery calcification, FH: family history, JNC_V: joint national committee, NCEP: national cholesterol education program, PB: blood pressure, OGTT: oral glucose tolerance test, hs Troponin: high-sensitivity Troponin I, MetS: metabolic syndrome, CCA: common carotid artery, AA: African American, LAT: Latinos, AS: Asian, BMI: body mass index.

Reference list of the included studies:

1. Iribarren C, Lu M, Jorgenson E, Martínez M, Lluis-Ganella C, Subirana I, et al. Clinical Utility of Multimarker Genetic Risk Scores for Prediction of Incident Coronary Heart Disease: A Cohort Study Among Over 51 000 Individuals of European Ancestry. Circulation Cardiovascular genetics. 2016;9(6):531-40.
2. Hughes MF, Saarela O, Stritzke J, Kee F, Silander K, Klopp N, et al. Genetic markers enhance coronary risk prediction in men: the MORGAM prospective cohorts. PloS one. 2012;7(7):e40922-e.
3. Talmud PJ, Cooper JA, Palmen J, Lovering R, Drenos F, Hingorani AD, et al. Chromosome 9p21.3 Coronary Heart Disease Locus Genotype and Prospective Risk of CHD in Healthy Middle-Aged Men. Clinical Chemistry. 2008;54(3):467-74.
4. Humphries SE, Cooper JA, Talmud PJ, Miller GJ. Candidate Gene Genotypes, Along with Conventional Risk Factor Assessment, Improve Estimation of Coronary Heart Disease Risk in Healthy UK Men. Clinical Chemistry. 2007;53(1):8-16.
5. Beaney KE, Cooper JA, Drenos F, Humphries SE. Assessment of the clinical utility of adding common single nucleotide polymorphism genetic scores to classical risk factor algorithms in coronary heart disease risk prediction in UK men. Clinical chemistry and laboratory medicine. 2017;55(10):1605-13.
6. Antiochos P, Marques-Vidal P, McDaid A, Waeber G, Vollenweider P. Association between parental history and genetic risk scores for coronary heart disease prediction: The population-based CoLaus study. Atherosclerosis. 2016; 244:59-65.
7. Brautbar A, Pompeii LA, Dehghan A, Ngwa JS, Nambi V, Virani SS, et al. A genetic risk score based on direct associations with coronary heart disease improves coronary heart disease risk prediction in the Atherosclerosis Risk in Communities (ARIC), but not in the Rotterdam and Framingham Offspring, Studies. Atherosclerosis. 2012;223(2):421-6.
8. Chien KL, Hsu HC, Su TC, Chen MF, Lee YT, Hu FB. Apolipoprotein B and non-high density lipoprotein cholesterol and the risk of coronary heart disease in Chinese. Journal of lipid research. 2007;48(11):2499-505.
9. Simmons RK, Sharp S, Boekholdt SM, Sargeant LA, Khaw KT, Wareham NJ, et al. Evaluation of the Framingham risk score in the European Prospective Investigation of Cancer-Norfolk cohort: does adding glycated hemoglobin improve the prediction of coronary heart disease events? Archives of internal medicine. 2008;168(11):1209-16.
10. Macleod J, Metcalfe C, Smith GD, Hart C. Does consideration of either psychological or material disadvantage improve coronary risk prediction? Prospective observational study of Scottish men. J Epidemiol Community Health. 2007;61(9):833-7.
11. Nishimura K, Okamura T, Watanabe M, Nakai M, Takegami M, Higashiyama A, et al. Predicting Coronary Heart Disease Using Risk Factor Categories for a Japanese Urban Population, and Comparison with the Framingham Risk Score: The Suita Study. Journal of atherosclerosis and thrombosis. 2016;23(9):1138-9.
12. Ingelsson E, Schaefer EJ, Contois JH, McNamara JR, Sullivan L, Keyes MJ, et al. Clinical Utility of Different Lipid Measures for Prediction of Coronary Heart Disease in Men and Women. Jama. 2007;298(7):776-85.
13. Cao J, Steffen BT, Guan W, Remaley AT, McConnell JP, Palamalai V, et al. A comparison of three apolipoprotein B methods and their associations with incident coronary heart disease risk over a 12-year follow-up period: The Multi-Ethnic Study of Atherosclerosis. Journal of Clinical Lipidology. 2018;12(2):300-4.
14. Nambi V, Chambless L, Folsom AR, He M, Hu Y, Mosley T, et al. Carotid Intima-Media Thickness and Presence or Absence of Plaque Improves Prediction of Coronary Heart Disease Risk: The ARIC (Atherosclerosis Risk In Communities) Study. Journal of the American College of Cardiology. 2010;55(15):1600-7.
15. Cushman M, Arnold AM, Psaty BM, Manolio TA, Kuller LH, Burke GL, et al. C-Reactive Protein and the 10-Year Incidence of Coronary Heart Disease in Older Men and Women. Circulation. 2005;112(1):25-31.

16. Cooper JA, Miller GJ, Humphries SE. A comparison of the PROCAM and Framingham point-scoring systems for estimation of individual risk of coronary heart disease in the Second Northwick Park Heart Study. Atherosclerosis. 2005;181(1):93-100.
17. Orford JL, Sesso HD, Stedman M, Gagnon D, Vokonas P, Gaziano JM. A comparison of the Framingham and European society of cardiology coronary heart disease risk prediction models in the normative aging study. American Heart Journal. 2002;144(1):95-100.
18. Jee SH, Jang Y, Oh DJ, Oh B-H, Lee SH, Park S-W, et al. A coronary heart disease prediction model: the Korean Heart Study. BMJ Open. 2014;4(5):e005025.
19. Merry AH, Boer JM, Schouten LJ, Ambergen T, Steyerberg EW, Feskens EJ, et al. Risk prediction of incident coronary heart disease in The Netherlands: re-estimation and improvement of the SCORE risk function. European journal of preventive cardiology. 2012;19(4):840-8.
20. Khalili D, Hadaegh F, Fahimfar N, Shafiee G, Sheikholeslami F, Ghanbarian A, et al. Does an electrocardiogram add predictive value to the rose angina questionnaire for future coronary heart disease? 10-year follow-up in a Middle East population. J Epidemiol Community Health. 2012;66(12):1104-9.
21. Auer R, Bauer DC, Marques-Vidal P, Butler J, Min LJ, Cornuz J, et al. Association of major and minor ECG abnormalities with coronary heart disease events. Jama. 2012;307(14):1497-505.
22. Taylor AJ, Feuerstein I, Wong H, Barko W, Brazaitis M, O'Malley PG. Do conventional risk factors predict subclinical coronary artery disease? Results from the Prospective Army Coronary Calcium Project. American Heart Journal. 2001;141(3):463-8.
23. Rana JS, Cote M, Després JP, Sandhu MS, Talmud PJ, Ninio E, et al. Inflammatory biomarkers and the prediction of coronary events among people at intermediate risk: the EPIC-Norfolk prospective population study. Heart. 2009;95(20):1682-7.
24. Parikh NI, Jeppson RP, Berger JS, Eaton CB, Kroenke CH, LeBlanc ES, et al. Reproductive Risk Factors and Coronary Heart Disease in the Women’s Health Initiative Observational Study. Circulation. 2016;133(22):2149-58.
25. De Vries PS, Kavousi M, Ligthart S, Uitterlinden AG, Hofman A, Franco OH, et al. Incremental predictive value of 152 single nucleotide polymorphisms in the 10-year risk prediction of incident coronary heart disease: the Rotterdam Study. Int J Epidemiol. 2015;44(2):682-8.
26. Paynter NP, Crainiceanu CM, Sharrett AR, Chambless LE, Coresh J. Effect of correcting for long-term variation in major coronary heart disease risk factors: relative hazard estimation and risk prediction in the Atherosclerosis Risk in Communities Study. Ann Epidemiol. 2012;22(3):191-7.
27. Morrison AC, Bare LA, Chambless LE, Ellis SG, Malloy M, Kane JP, et al. Prediction of Coronary Heart Disease Risk using a Genetic Risk Score: The Atherosclerosis Risk in Communities Study. American Journal of Epidemiology. 2007;166(1):28-35.
28. Folsom AR, Chambless LE, Ballantyne CM, Coresh J, Heiss G, Wu KK, et al. An assessment of incremental coronary risk prediction using C-reactive protein and other novel risk markers: the atherosclerosis risk in communities study. Archives of internal medicine. 2006;166(13):1368-73.
29. Detrano RC, Wong ND, Doherty TM, Shavelle RM, Tang W, Ginzton LE, et al. Coronary calcium does not accurately predict near-term future coronary events in high-risk adults. Circulation. 1999;99(20):2633-8.
30. Tada H, Melander O, Louie JZ, Catanese JJ, Rowland CM, Devlin JJ, et al. Risk prediction by genetic risk scores for coronary heart disease is independent of self-reported family history. European Heart Journal. 2015;37(6):561-7.
31. Aekplakorn W, Pakpeankitwatana V, Lee CMY, Woodward M, Barzi F, Yamwong S, et al. Abdominal Obesity and Coronary Heart Disease in Thai Men. Obesity. 2007;15(4):1036-42.
32. Bolton JL, Stewart MCW, Wilson JF, Anderson N, Price JF. Improvement in prediction of coronary heart disease risk over conventional risk factors using SNPs identified in genome-wide association studies.PloSone.2013;8(2):e57310-e.

33. Lloyd-Jones DM, Wilson PW, Larson MG, Beiser A, Leip EP, D'Agostino RB, et al. Framingham risk score and prediction of lifetime risk for coronary heart disease. The American journal of cardiology. 2004;94(1):20-4.
34. Empana JP, Ducimetière P, Arveiler D, Ferrières J, Evans A, Ruidavets JB, et al. Are the Framingham and PROCAM coronary heart disease risk functions applicable to different European populations?: The PRIME Study. European Heart Journal. 2003;24(21):1903-11.
35. Rodondi N, Locatelli I, Aujesky D, Butler J, Vittinghoff E, Simonsick E, et al. Framingham Risk Score and Alternatives for Prediction of Coronary Heart Disease in Older Adults. PloS one. 2012;7(3):e34287.
36. McGeechan K, Liew G, Macaskill P, Irwig L, Klein R, Sharrett AR, et al. Risk Prediction of Coronary Heart Disease Based on Retinal Vascular Caliber (from the Atherosclerosis Risk In Communities [ARIC] Study). The American journal of cardiology. 2008;102(1):58-63.
37. Onat A, Dursunoğlu D, Sansoy V. Relatively high coronary death and event rates in Turkish women: Relation to three major risk factors in five-year follow-up of cohort. International Journal of Cardiology. 1997;61(1):69-77.
38. Wilson PW, D'Agostino RB, Levy D, Belanger AM, Silbershatz H, Kannel WB. Prediction of coronary heart disease using risk factor categories. Circulation. 1998;97(18):1837-47.
39. Mainous AG, 3rd, Everett CJ, Player MS, King DE, Diaz VA. Importance of a patient's personal health history on assessments of future risk of coronary heart disease. Journal of the American Board of Family Medicine : JABFM. 2008;21(5):408-13.
40. Pyörälä M, Miettinen H, Laakso M, Pyörälä K. Hyperinsulinemia Predicts Coronary Heart Disease Risk in Healthy Middle-aged Men. Circulation. 1998;98(5):398-404.
41. Thomsen TF, McGee D, Davidsen M, Jørgensen T. A cross-validation of risk-scores for coronary heart disease mortality based on data from the Glostrup Population Studies and Framingham Heart Study. International Journal of Epidemiology. 2002;31(4):817-22.
42. Bye A, Røsjø H, Nauman J, Silva GJJ, Follestad T, Omland T, et al. Circulating microRNAs predict future fatal myocardial infarction in healthy individuals – The HUNT study. Journal of Molecular and Cellular Cardiology. 2016; 97:162-8.
43. Kavousi M, Elias-Smale S, Rutten JHW, Leening MJG, Vliegenthart R, Verwoert GC, et al. Evaluation of newer risk markers for coronary heart disease risk classification: a cohort study. Ann Intern Med. 2012;156(6):438-44.
44. Ganna A, Magnusson PK, Pedersen NL, de Faire U, Reilly M, Arnlöv J, et al. Multilocus genetic risk scores for coronary heart disease prediction. Arteriosclerosis, thrombosis, and vascular biology. 2013;33(9):2267-72.
45. Cooper JA, Miller GJ, Bauer KA, Morrissey JH, Meade TW, Howarth DJ, et al. Comparison of Novel Hemostatic Factors and Conventional Risk Factors for Prediction of Coronary Heart Disease. Circulation. 2000;102(23):2816-22.
46. Arima H, Kubo M, Yonemoto K, Doi Y, Ninomiya T, Tanizaki Y, et al. High-sensitivity C-reactive protein and coronary heart disease in a general population of Japanese: the Hisayama study. Arteriosclerosis, thrombosis, and vascular biology. 2008;28(7):1385-91.
47. Brautbar A, Ballantyne CM, Lawson K, Nambi V, Chambless L, Folsom AR, et al. Impact of Adding a Single Allele in the 9p21 Locus to Traditional Risk Factors on Reclassification of Coronary Heart Disease Risk and Implications for Lipid-Modifying Therapy in the Atherosclerosis Risk in Communities Study. Circulation: Cardiovascular Genetics. 2009;2(3):279-85.
48. St-Pierre AC, Cantin B, Dagenais GR, Després J-P, Lamarche B. Apolipoprotein-B, Low-Density Lipoprotein Cholesterol, and the Long-Term Risk of Coronary Heart Disease in Men. The American journal of cardiology. 2006;97(7):997-1001.
49. Ryoo J-H, Park SK, Hong HP, Kim M-G, Ha CS. Clinical significance of serum apolipoproteins as a predictor of coronary heart disease risk in Korean men. Clinical Endocrinology. 2016;84(1):63-71.

50. Yarnell JWG, Patterson CC, Sweetnam PM, Lowe GDO. Haemostatic/inflammatory markers predict 10-year risk of IHD at least as well as lipids: the Caerphilly collaborative studies. European Heart Journal. 2004;25(12):1049-56.
51. Everage NJ, Gjelsvik A, McGarvey ST, Linkletter CD, Loucks EB. Inverse Associations Between Perceived Racism and Coronary Artery Calcification. Ann Epidemiol. 2012;22(3):183-90.
52. Iribarren C, Chandra M, Rana JS, Hlatky MA, Fortmann SP, Quertermous T, et al. High-sensitivity cardiac troponin I and incident coronary heart disease among asymptomatic older adults. Heart. 2016;102(15):1177-82.
53. McClelland RL, Jorgensen NW, Budoff M, Blaha MJ, Post WS, Kronmal RA, et al. 10-Year Coronary Heart Disease Risk Prediction Using Coronary Artery Calcium and Traditional Risk Factors: Derivation in the MESA (Multi-Ethnic Study of Atherosclerosis) With Validation in the HNR (Heinz Nixdorf Recall) Study and the DHS (Dallas Heart Study). Journal of the American College of Cardiology. 2015;66(15):1643-53.
54. Liu J, Hong Y, D'Agostino S, Ralph B., Wu Z, Wang W, Sun J, et al. Predictive Value for the Chinese Population of the Framingham CHD Risk Assessment Tool Compared With the Chinese Multi-provincial Cohort Study. Jama. 2004;291(21):2591-9.
55. Brant LJ, Ferrucci L, Sheng SL, Concin H, Zonderman AB, Kelleher CC, et al. Gender differences in the accuracy of time-dependent blood pressure indices for predicting coronary heart disease: A random-effects modeling approach. Gender Medicine. 2010;7(6):616-27.
56. Onat A, Can G, Hergenç G, Uğur M, Yüksel H. Coronary disease risk prediction algorithm warranting incorporation of C-reactive protein in Turkish adults, manifesting sex difference. Nutrition, Metabolism and Cardiovascular Diseases. 2012;22(8):643-50.
57. Cross DS, McCarty CA, Hytopoulos E, Beggs M, Nolan N, Harrington DS, et al. Coronary risk assessment among intermediate risk patients using a clinical and biomarker-based algorithm developed and validated in two population cohorts. Curr Med Res Opin. 2012;28(11):1819-30.
58. Hadaegh F, Mohebi R, Bozorgmanesh M, Saadat N, Sheikholeslami F, Azizi F. Electrocardiographic abnormalities improve classification of coronary heart disease risk in women: Tehran Lipid and Glucose Study. Atherosclerosis. 2012;222(1):110-5.
59. Kang HM, Kim D-J. Metabolic Syndrome versus Framingham Risk Score for Association of Self-Reported Coronary Heart Disease: The 2005 Korean Health and Nutrition Examination Survey. Diabetes Metab J. 2012;36(3):237-44.
60. Kivimäki M, Nyberg ST, Batty GD, Shipley MJ, Ferrie JE, Virtanen M, et al. Does adding information on job strain improve risk prediction for coronary heart disease beyond the standard Framingham risk score? The Whitehall II study. International journal of epidemiology. 2011;40(6):1577-84.
61. Gander JC, Sui X, Hébert JR, Hazlett LJ, Cai B, Lavie CJ, et al. Association of Cardiorespiratory Fitness With Coronary Heart Disease in Asymptomatic Men. Mayo Clinic Proceedings. 2015;90(10):1372-9.
62. Arad Y, Goodman KJ, Roth M, Newstein D, Guerci AD. Coronary Calcification, Coronary Disease Risk Factors, C-Reactive Protein, and Atherosclerotic Cardiovascular Disease Events: The St. Francis Heart Study. Journal of the American College of Cardiology. 2005;46(1):158-65.
63. Pischon T, Girman CJ, Sacks FM, Rifai N, Stampfer MJ, Rimm EB. Non-high-density lipoprotein cholesterol and apolipoprotein B in the prediction of coronary heart disease in men. Circulation. 2005;112(22):3375-83.
64. Polak JF, Szklo M, O'Leary DH. Associations of Coronary Heart Disease with Common Carotid Artery Near and Far Wall Intima-Media Thickness: The Multi-Ethnic Study of Atherosclerosis. Journal of the American Society of Echocardiography. 2015;28(9):1114-21.
65. Cavus E, Karakas M, Ojeda FM, Kontto J, Veronesi G, Ferrario MM, et al. Association of Circulating Metabolites With Risk of Coronary Heart Disease in a European Population: Results From the Biomarkers for Cardiovascular Risk Assessment in Europe (BiomarCaRE) Consortium. JAMA cardiology. 2019;4(12):1270-9.

66. Subirana I, Fitó M, Diaz O, Vila J, Francés A, Delpon E, et al. Prediction of coronary disease incidence by biomarkers of inflammation, oxidation, and metabolism. Scientific reports. 2018;8(1):3191.
67. Hindy G, Wiberg F, Almgren P, Melander O, Orho-Melander M. Polygenic Risk Score for Coronary Heart Disease Modifies the Elevated Risk by Cigarette Smoking for Disease Incidence. Circulation: Genomic and Precision Medicine. 2018;11(1): e001856.
68. Chien KL, Lin HJ, Su TC, Chen YY, Chen PC. Comparing the Consistency and Performance of Various Coronary Heart Disease Prediction Models for Primary Prevention Using a National Representative Cohort in Taiwan. Circulation journal: official journal of the Japanese Circulation Society. 2018;82(7):1805-12.
69. Iribarren C, Lu M, Jorgenson E, Martínez M, Lluis-Ganella C, Subirana I, et al. Weighted Multi-marker Genetic Risk Scores for Incident Coronary Heart Disease among Individuals of African, Latino and East-Asian Ancestry. Scientific reports. 2018;8(1):6853.
70. Can G, Onat A, Sayılı U, Hayıroğlu M, Ademoglu E, Yurtseven E. Optimal anthropometric measures to predict incidence of coronary heart disease in adults in Turkey. The National medical journal of India. 2019; 32:334-41.
71. Wang Z, Zhu C, Nambi V, Morrison AC, Folsom AR, Ballantyne CM, et al. Metabolomic Pattern Predicts Incident Coronary Heart Disease. Arteriosclerosis, thrombosis, and vascular biology. 2019;39(7):1475-82.
72. Cao J, Steffen BT, Guan W, Remaley AT, McConnell JP, Palamalai V, et al. A comparison of three apolipoprotein B methods and their associations with incident coronary heart disease risk over a 12-year follow-up period: The Multi-Ethnic Study of Atherosclerosis. Journal of Clinical Lipidology. 2018;12(2):300-4.
